# Whole-exome and targeted sequencing identify *ROBO1* and *ROBO2* mutations as progression-related drivers in myelodysplastic syndromes

**DOI:** 10.1038/ncomms9806

**Published:** 2015-11-26

**Authors:** Feng Xu, Ling-Yun Wu, Chun-Kang Chang, Qi He, Zheng Zhang, Li Liu, Wen-Hui Shi, Juan Guo, Yang Zhu, You-Shan Zhao, Shu-Cheng Gu, Cheng-Ming Fei, Dong Wu, Li-Yu Zhou, Ji-Ying Su, Lu-Xi Song, Chao Xiao, Xiao Li

**Affiliations:** 1 Department of Hematology, Shanghai Jiao Tong University Affiliated Sixth People's Hospital, Shanghai 200233, China

## Abstract

The progressive mechanism underlying myelodysplastic syndrome remains unknown. Here we identify *ROBO1* and *ROBO2* as novel progression-related somatic mutations using whole-exome and targeted sequencing in 6 of 16 (37.5%) paired MDS patients with disease progression. Further deep sequencing detects 20 (10.4%) patients with *ROBO* mutations in a cohort of 193 MDS patients. In addition, copy number loss and loss of heterogeneity (LOH) of *ROBO1* and *ROBO2* are frequently observed in patients with progression or carrying *ROBO* mutations. In *in vitro* experiments, overexpression of ROBO1 or ROBO2 produces anti-proliferative and pro-apoptotic effects in leukaemia cells. However, this effect was lost in ROBO mutants and ROBO-SLIT2 signalling is impaired. Multivariate analysis shows that *ROBO* mutations are independent factors for predicting poor survival. These findings demonstrate a novel contribution of *ROBO* mutations to the pathogenesis of MDS and highlight a key role for ROBO-SLIT2 signalling in MDS disease progression.

## Abstract (web summary)

The molecular events that drive the disease progression of myelodysplastic syndromes are poorly understood. Here, the authors report the identification of novel progression-related somatic mutations in *ROBO1* and *ROBO2*, highlighting ROBO-SLIT2 signalling in the pathogenesis of MDS.

## Graphical abstract

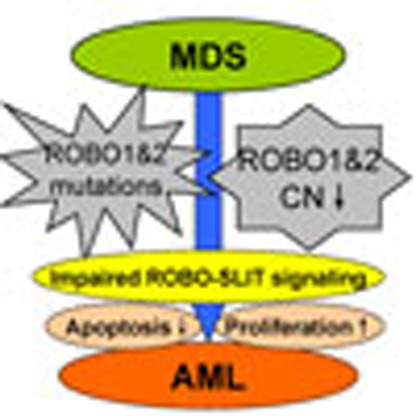

Myelodysplastic syndromes (MDSs) are a heterogeneous group of haematopoietic stem cell disorders characterized by ineffective haematopoiesis and peripheral blood cytopenias^1^. Up to 30% of individuals with MDS will progress to acute myeloid leukaemia (AML)^2^. Although a patient with MDS could remain at the lower risk stage, newly emerging events or an incremental burden of pre-existing events may cause rapid progression to a higher risk stage, resulting in AML. Thus, there is a compelling need to identify the specific molecular events (driving events) that promote this transformation.

In recent years, whole-genome or -exome sequencing technologies have been successively applied to identify massive genetic alterations in MDS^3^,^4^,^5^,^6^,^7^. These alterations are involved in several functional gene categories, including the RNA splicing machinery, epigenetic effectors, cohesin/cell adhesion and cell signalling^6^,^7^. Some gene mutations, such as those in *ASXL1*, *RUNX1*, *TET2*, *IDH1*, *IDH2* and *SETBP1*, have been considered to be partially responsibility for disease progression in MDS based on the evolutions of mutations in the same individuals whose disease had progression^5^,^8^. However, these progression-related mutations remain to be confirmed in other studies. One study indicated that *ASXL1* mutations may contribute little to disease progression^9^, although the prominent role of ASXL1 in MDS development has been explicitly depicted *in vivo*^10^,^11^,^12^. To gain new insight into the molecular mechanism underlying disease progression in MDS, we conducted whole-exome and targeted sequencing of serial bone marrow samples from patients with disease progression and attempted to discover progression-related somatic mutations.

In this study, using whole-exome and targeted sequencing, we identify roundabout guidance receptor 1 (*ROBO1*) and *ROBO2* mutations as progression-related drivers in MDS. Next-generation sequencing reveals that 20 (10.4%) of 193 MDS patients carry the *ROBO1* or *ROBO2* mutations. Overexpression of ROBO1 or ROBO2 produces anti-proliferative and pro-apoptotic effects in leukaemia cells *in vitro*. *ROBO* mutations are independent factors for predicting poor survival. The present results revealed a novel contribution to the mutation profile of MDS and suggests that it plays a role in MDS disease progression.

## Results

### Whole-exome sequencing of three paired MDS cases

Whole-exome sequencing was performed in three paired samples of MDS cases (Supplementary Table 1). The bone marrow samples were obtained at the lower risk stage (time of diagnosis) and higher risk stage (disease progression) with matched oral mucosal epithelial samples. The average target coverage was 53 ×. Of these, 89.0% of the reads had a Phred-like quality score (*Q* score) greater than 20, and 78.4% of the reads had a *Q* score greater than 30. The proportion of target bases with read depths of 2 ×, 10 ×, 20 × and 30 × was 92.4%, 82.2%, 68.9% and 56.3%, respectively (Supplementary Table 2). We screened all of the single-nucleotide variants (SNVs) by comparing the variants identified in the bone marrow exome data set with the 1000-g database (frequency threshold <0.001) and germline variants present in the oral mucosal epithelial samples (Supplementary Table 3 and Supplementary Data 1). We identified 507 potential somatic sequence changes (identification flow described in Supplementary Fig. 1).

**Figure 1:**
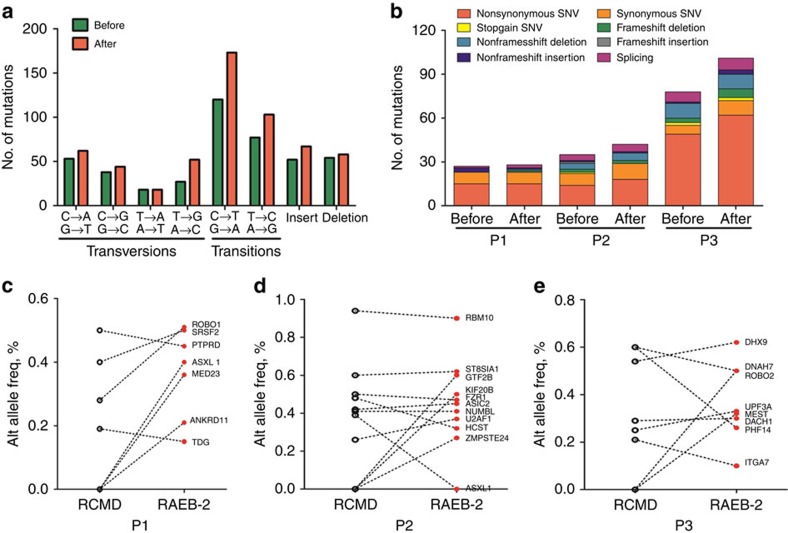
Whole-exome sequencing of three paired MDS at the lower and higher risk stages. (**a**) The nucleotide substitutions for somatically acquired point mutations are shown in three paired MDS before and after disease progression. C→T/G→A transitions were the primary nucleotide changes. (**b**) Distribution of the numbers and categories of somatically acquired point mutations before and after disease progression. The number of nonsynonymous SNVs was predominant and increased after disease progression. (**c**–**e**) The altered allele frequency (Alt allele Freq.) of a series of gene mutations in three paired MDS before and after disease progression.

For the nucleotide substitution, the mutation spectrum showed a predominance of C→T/G→A transitions (36.0–38.3%), followed by A→G/T→C transitions (22.8–23.1%) and G→T/C→A transversions (13.7–15.9%) before or after disease progression (Fig. 1a). This mutation spectrum is similar to those reported in gastrointestinal cancers, leukaemia and MDS^6^,^13^,^14^, but it differs from those observed in lung cancer with a prevalence of G→T/C→A transversions^15^,^16^. The number of multiple nucleotide substitutions, particularly C→T/G→A transitions, T→G/A→C transversions and A→G/T→C transitions, increased to varying degrees from a lower risk to a higher risk stage. For these types of mutation that affect the protein-coding sequence (CDS), the number of non-synonymous SNVs was advantageous and increased after disease progression (Patient 3 (P3); Fig. 1b).

We focused on those changes that were predicted to affect the protein-CDS, including 69 non-synonymous substitutions and 15 insertion or deletions (indels; Supplementary Data 2). Considering that each read of a massively parallel sequencing corresponded to a single molecule of genomic DNA (gDNA), the proportion of sequencing reads reporting a variant allele provides a quantitative estimate of the mutation burden. As shown in Fig. 1c–e, the variability of an altered allele frequency in a mutant allele was clearly greater (0.10–0.94) before or after disease progression. For Patient 1 (P1), the appearance of new somatic mutations such as *ASXL1*, *MED23* and *ANKRD11* as well as the incremental burden of the *ROBO1* mutation should be considered as key drivers of disease progression at a higher risk stage. For Patient 2 (P2) and Patient 3 (P3), emerging mutations, including *GTF2B*, *KIF20B*, *ZMPSTE24*, *DNAH7* and *MEST*, may promote disease progression. After disease progression, most of the original somatic mutations associated with the lower risk stage remain present with an increase or decrease in frequency of altered allele. These data suggest that the population of malignant cells in MDS is commonly genetically heterogeneous, with some emerging mutations or increased mutant burden associated with disease progression.

Based on the following choice criteria of gene recurrence, high priority, cancer relevance and new presence in disease progression, 26 marker genes (*ANKRD11*, *ASIC2*, *ASXL1*, *DACH1*, *DHX9*, *FZR1*, *GTF2B*, *HCST*, *ITIH3*, *KIF20B*, *MED23*, *MEST*, *NUMBL*, *PHF14*, *PTPRD*, *RBM10*, *ROBO1*, *ROBO2*, *SRSF2*, *ST8SIA1*, *TDG*, *U2AF1*, *UPD3A*, *ZMPSTE24*, *EZH2* and *SF3B1*) were screened for targeted next-generation sequencing (detailed mutation information is shown in Supplementary Table 4). Among these genes, we identified a somatic *ROBO1* (p.R116H) mutation in P1 and *ROBO2* (p. R522Q) mutation in P3. For verification, we amplified the corresponding genomic region directly from the original samples using PCR and Sanger sequencing. Sanger sequencing confirmed that the *ROBO1* and *ROBO2* mutations were somatic changes (Supplementary Fig. 2).

### Validated sequencing in additional 13 paired patients

**Figure 2:**
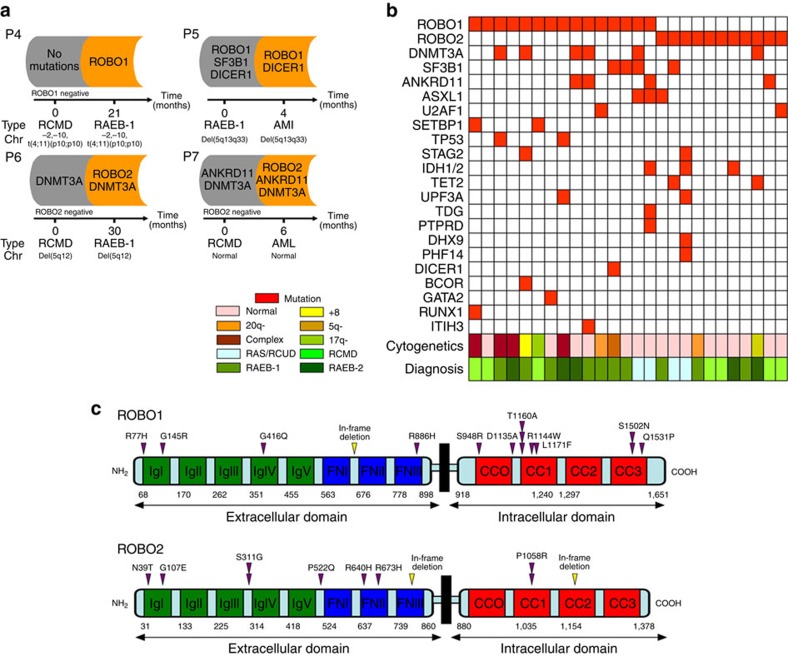
Targeted sequencing of the *ROBO1* and *ROBO2* genes. (**a**) Profiles of gene mutations in four representative cases with MDS that underwent transformed to RAEB-1 (Patient 4 and Patient 6) or acute myeloid leukaemia (AML; Patient 5 and Patient 7). (**b**) The relationship of *ROBO* mutations with other common mutations. Coexisting mutations in the *ROBO1*- or *ROBO2*-mutated cohort are shown in a matrix: 20 of 26 cases (76.9%) were positive for other concomitant somatic mutations. (**c**) *ROBO1* and *ROBO2* somatic non-synonymous alterations (inverted triangles) are depicted over the affected protein domains. IgI–V (immunoglobulin-like domains) and FNI–III (fibronectin-type domains) belong to the extracellular domain; CC0–3 (conserved cytoplasmic motifs) constitutes the intracellular domain. RAEB-1 or 2, refractory anemia with excess blasts 1 or 2; RARS, refractory anemia with ring sideroblasts; RCMD, refractory cytopenia with multilineage dysplasia.

The presence of identical mutations that were not previously involved in MDS in two different cases in which disease had progressed prompted us to validate the mutations in *ROBO1* and *ROBO2* in samples from additional subjects with paired samples at both lower and higher risk stages. In addition, to identify whether the other mutated genes in MDS emerged with disease progression, 24 genes screened from the whole-exome sequencing mutation library and 13 known genes (*DNMT3A*, *TET2*, *STAG2*, *SETBP1*, *IDH1*, *IDH2*, *WT1*, *TP53*, *CEBPA*, *GATA2*, *RUNX1*, *BCOR* and *ZRSR2*) that are frequently mutated in MDS were also target sequenced using next-generation sequencing. An additional 13 paired MDS samples from patients at lower and higher risk stages were subjected to targeted sequencing (Supplementary Tables 5 and 6). Targeted deep sequencing (average sufficient depth>100; sequencing information shown in Supplementary Data 3) revealed that 4 out of 13 (30.8%) patients acquired a *ROBO1* (*n*=2) or *ROBO2* (*n*=2) mutation at the disease progression stage (Fig. 2a and Supplementary Table 6). One *ROBO1* mutation (p.T1160A) was found in a patient with refractory anemia with excess blast (RAEB)-1 that was not detected at the refractory cytopenia with multilineage dysplasia (RCMD) stage. The other *ROBO1* mutation (p.R416Q) was observed in a patient with RAEB-1 who rapidly evolved to AML. Two patients acquired a *ROBO2* mutation (p.G107E and p.R640H) during their progression to the RAEB-1 and AML stages, respectively. In a comprehensive analysis combined with the data obtained from whole-exome sequencing, 6 out of 16 (37.5%) pairs of patients acquired the *ROBO1* (*n*=3) or *ROBO2* (*n*=3) mutation at the disease progression stage. Interestingly, mutual exclusivity could be observed between *ROBO1* and *ROBO2*, probably because they are in the same functional categories. We also detected *SRSF2* and *ASXL1* mutations in 5 (31.3%) and DNMT3A mutations in 3(18.8%) of 16 pairs of patients before and after the disease progression stage (Supplementary Table 6). Previous have shown that *SRSF2*, *ASXL1* and *DNMT3A* mutations predict a poor prognosis^6^,^7^,^17^,^18^. *ROBO1*, *ROBO2*, *SRSF2*, *ASXL1* and *DNMT3A* mutations may contribute to the disease progression of MDS.

### Deep sequencing of the *ROBO* mutations in 193 MDS cases

To further analyse the mutation frequency of *ROBO1* and *ROBO2*, we performed targeted deep sequencing in a cohort of 193 patients with primary MDS who received no treatment (Supplementary Table 7). Among the 193 MDS patients, 12 cases displayed *ROBO1* mutations and 8 cases had *ROBO2* mutations. Taken together with the data from the 16 pairs of patients, we detected 26 (12.4%) *ROBO1* and *ROBO2* mutations (*ROBO1*, *n*=15; *ROBO2*, *n*=11) in 209 patients with MDS. Details regarding the *ROBO* mutations and sequencing information are shown in Supplementary Table 8 and Supplementary Data 4. All of these mutations were further verified by Sanger sequencing (Supplementary Fig. 3). DNA from matched oral mucosal epithelial samples was available for 8 of the 26 patients with *ROBO1* or *ROBO2* mutations. Sanger sequencing of germline controls confirmed that these mutations were somatic changes (Supplementary Fig. 4). In addition, we performed snapshot sequencing in 100 normal older adults with a median age of 60 years. The results showed that none of the 26 *ROBO1* or *ROBO2* mutations were present in these normal controls (Supplementary Data 5).

These mutations were dispersed throughout the majority of the entire coding region (Fig. 2c). Among the 26 *ROBO1* and *ROBO2* mutations, 23 were missense mutations and 3 were splicing mutations. In terms of the mutation distribution, ∼70% of the *ROBO1* mutations were located in the intracellular domain, whereas more than 70% of the *ROBO2* mutations were located in the extracellular domain. In addition, the distribution of *ROBO1* and *ROBO2* mutations was heterogeneous and there were no mutation hot spots, although the incidence of the p.T1160A mutant was relatively frequent. The SIFT Score in the majority of the *ROBO* mutations (22/26, 84.6%) was less than 0.05, indicating that the *ROBO* mutations may have a serious effect on the function of protein.

We also identified other gene mutations in 193 cases with MDS. Pooled with the data from 15 pairs of patients, several genes exhibited a relative high frequency of occurrence, including *ASXL1* (12.9%, 27/209), *TET2* (11.5%, 24/209), *DHX9* (9.6%, 20/209), *DNMT3A* (8.1%, 17/209), *IDH1* and *IDH2* (7.2%, 15/209), *SF3B1* (6.7%, 14/209), *ANKRD11* (6.2%, 13/209), *SRSF2* (5.7%, 12/209), *U2AF1* (5.7%, 12/209), *STAG2* (5.3%, 11/209) and *SETBP1* (5.3%, 11/209; Supplementary Fig. 5). Mutual exclusivity was again observed between *ROBO1* and *ROBO2* (Fig. 2b). Regarding the relationship between *ROBO* mutations and other gene mutations, a *ROBO1* or *ROBO2* mutation with a *DNMT3A* mutation was observed in six patients. *ROBO1* or *ROBO2* with a *SF3B1* or *ANKRD11* mutation was detected in four patients, respectively. *ROBO1* or *ROBO2* with a *ASXL1* mutation was detected in three patients (Fig. 2b). However, the *ROBO* mutations appeared to demonstrate mutual exclusivity with several gene mutations such as *RUNX1*, *BCOR* and *GATA2*.

### Copy-number variation (CNV) analysis

**Figure 3:**
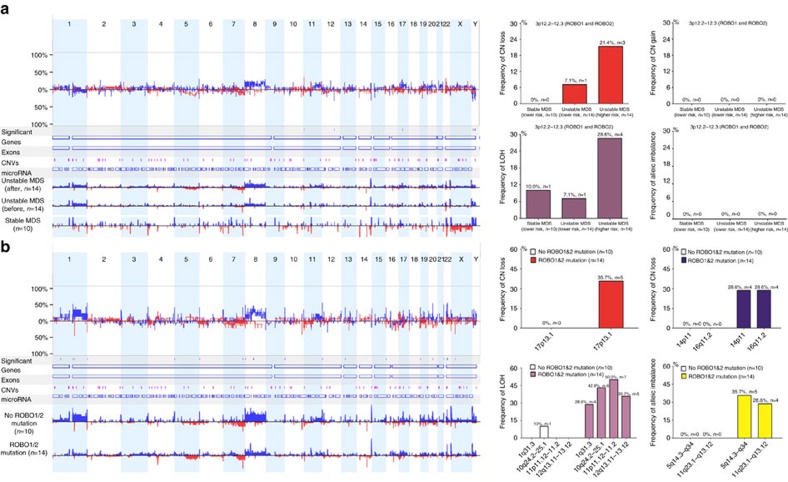
CNV and LOH analysis. (**a**) CNV analysis in unstable MDS with a lower risk stage (before progression, *n*=14), unstable MDS at a higher risk stage (after progression, *n*=14) and stable MDS at a lower risk stage (*n*=10). All of the unstable patients were from the same 14 cases despite the different disease stages. The results of the CNV and LOH analysis showed that the frequency of CNV and LOH in the *ROBO1* and *ROBO2* locus increased from the lower to the higher risk stages in 14 paired MDS patients. (**b**) The CNV and LOH between the patients with (*n*=14) and without (*n*=10) *ROBO* mutations were also compared. Those patients with *ROBO* mutations harboured several differential genomic changes, including CN loss in 17p13.1 (35.7% versus 0%), CN gain in 14p11 (28.6% versus 0%) and 16q11.2 (28.6% versus 0%) and allelic imbalance of 5q14.3-q34 (35.7% versus 0%).

**Figure 4:**
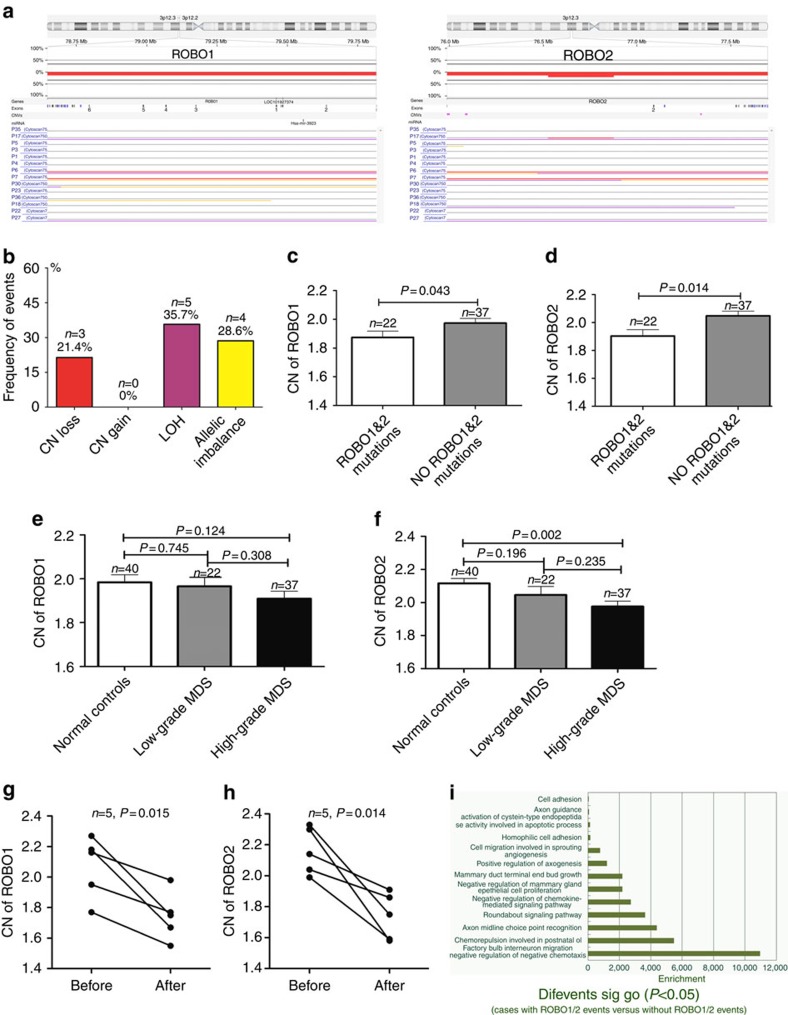
The relationship between *ROBO* mutations and CNV and LOH at the *ROBO1* and *ROBO2* locus. (**a**) The CNV and LOH events in the region of the *ROBO1* and *ROBO2* genes (3p12.2–12.3) are shown in 14 patients with *ROBO* mutations. Red line, copy number (CN) loss; purple line, loss of heterogeneity; yellow line, allelic imbalance. (**b**) Among 14 cases with *ROBO* mutations, 3 (21.4%) displayed CN loss, 5 (35.7%) had LOH and 4 (28.6%) showed an allelic imbalance at the *ROBO1* and *ROBO2* locus. Eight of fourteen cases exhibited genomic alterations. (**c**,**d**) The patients with *ROBO* mutations (*n*=22) exhibited significantly reduced CN in the *ROBO1* and *ROBO2* locus compared with patients without mutations (*n*=37; *P*=0.043; *P*=0.014). Statistical significance was determined by two-tailed Student's *t*-test. (**e**,**f**) The patients with high-grade MDS (*n*=37) exhibited reduced CN in the *ROBO1* and *ROBO2* locus compared with the normal controls (*n*=40; *P*=0.124; *P*=0.002). Statistical significance was determined by one-way ANOVA's LSD test. (**g**,**h**) The CN of *ROBO1* and *ROBO2* were clearly decreased after disease progression in five paired patients before and after disease progression (*n*=5; *P*=0.015; *P*=0.014). Statistical significance was determined by paired Student's *t* tests. (**i**) GO enrichment analysis according to CNV or LOH events in the *ROBO1* and *ROBO2* regions revealed that several cell biological behaviours, such as apoptosis, adhesion and proliferation, may be affected. Error bars throughout represent the s.e.m. The detection of ROBO1 or ROBO2 CN by quantitative reverse transcription–PCR was replicated for three times.

We employed a Cytosan 750K chip to analyse the CNVs and loss of heterogeneity (LOH) in patients with MDS. In general, a copy number (CN) gain in the chr8 region and CN loss or LOH in the chr 7 and chr 5 regions were the most common events (Supplementary Fig. 6). Next, we investigated the change in *ROBO1* and *ROBO2* genomic regions in several panels of patients. First, 38 patients were divided into three groups according to disease status: group 1, stable MDS with a lower risk (*n*=10) (sustained over 5 years at the lower risk stage); group 2, unstable MDS with a lower risk (before progression, *n*=14) and group 3, unstable MDS with a higher risk (after progression, *n*=14). Groups 2 and 3 were from the same 14 cases despite the different disease stages. The results showed that the frequency of CNV and LOH at the *ROBO1* and *ROBO2* locus increased from the lower (14.3%, 2/14) to the higher risk stage (50%, 7/14) in 14 paired MDS patients (Fig. 3a). Few CNVs and LOH events were observed in group 1 (only one LOH event in one patient). In addition to the genomic alteration at 3p12.2–12.3 (the locus of the *ROBO1* and *ROBO2* genes), a CN gain in chr 8 and CN loss in the chr 7 and chr 5 regions were more frequent with disease progression from the lower to the higher risk stage. Next, we compared the CNV and LOH between patients with and without a *ROBO* mutation (Fig. 3b). Those with a *ROBO* mutation harboured several differential genomic changes, including CN loss at 17p13.1 (35.7% versus 0%) and an allelic imbalance of 5q14.3-q34 (35.7% versus 0%). *TP53*, a tumour-suppressor gene located at 17p13.1, has been considered to play a vital role in leukaemia and MDS^19^,^20^. Finally, we focused on CNV and LOH events in the region of the *ROBO1* and *ROBO2* genes (3p12.2–12.3) in 14 patients carrying *ROBO* mutations. As shown in Fig. 4, among 14 cases with a *ROBO* mutation, 3 (21.4%) displayed CN loss, 5 (35.7%) had a LOH and 4 (28.6%) showed an allelic imbalance at the *ROBO1* and *ROBO2* locus (Fig. 4a,b). Eight of fourteen cases exhibited genomic alterations, indicating obvious instability at the *ROBO1* and *ROBO2* genomic locus in the mutated patients. To further validate the single-nucleotide polymorphism (SNP) array results, we performed quantitative genomic PCR for *ROBO1* and *ROBO2*. Based on the genomic quantitative PCR, the patients with *ROBO* mutations exhibited significantly reduced CN in the *ROBO1* and *ROBO2* locus compared with the patients without mutations (Student's *t*-test, *P*=0.043, *P*=0.014; Fig. 4c,d). In addition, the patients with high-grade MDS exhibited reduced CN in the *ROBO1* and *ROBO2* locus compared with the normal controls (one-way analysis of variance (ANOVA), least significance difference (LSD) test, *P*=0.124, *P*=0.002; Fig. 4e,f). Finally, we analysed the change in CN of *ROBO1* and *ROBO2* in five paired patients before and after disease progression. The results showed that the CN of *ROBO1* and *ROBO2* clearly decreased after disease progression (Fig. 4g,h). In addition, enrichment gene ontology (GO) analysis according to the CNV and LOH events in the *ROBO1* and *ROBO2* regions demonstrated that several cell biological behaviours, such as apoptosis, adhesion and proliferation, may be affected (Fig. 4i).

### Overexpression of ROBO1 or ROBO2 exerts anti-tumour effects

**Figure 5:**
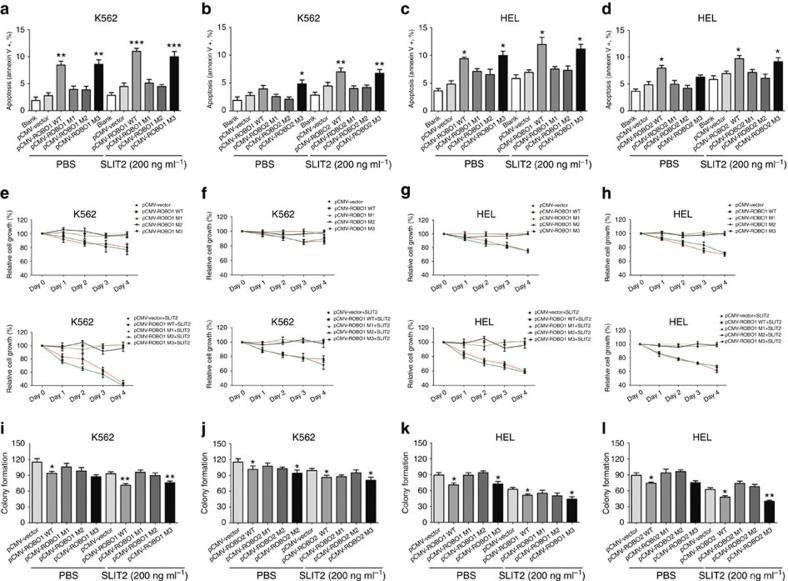
Overexpression of ROBO1 and ROBO2 inhibited tumorigenesis, whereas some ROBO mutants impaired ROBO function. Overexpression of wild-type ROBO1 and the ROBO1 R77H mutant (**a**,**c**), as well as wild-type ROBO2 and the ROBO2 G107E mutant (**b**,**d**), induced an increase in apoptosis in K562 (**a**,**b**) and HEL (**c**,**d**) cells. Exogenous SLIT2 (200 ng ml^−1^) enhanced the apoptotic effect. However, these effects were not observed in K562 and HEL cells overexpressing ROBO1 (T1160A and R886H) and ROBO2 (P1058R and P522Q) mutants. Overexpression of wild-type ROBO1 and the ROBO1 R77H mutant (**e**,**g**), as well as wild-type ROBO2 and the ROBO2 G107E mutant (**f**,**h**), inhibited cell growth in K562 (**e**,**f**) and HEL (**g**,**h**) cells (top). Exogenous SLIT2 (200 ng ml^−1^) enhanced the inhibitory effect on cell growth (bottom). However, the inhibitory effects were not obvious in K562 and HEL cells overexpressing ROBO1 (T1160A and R886H) and ROBO2 (P1058R and P522Q) mutants. In addition, overexpression of wild-type ROBO1 and the ROBO1 R77H mutant (**i**,**k**), as well as wild-type ROBO2 and the ROBO2 G107E mutant (**j**,**l**), inhibited colony formation in K562 (**i**,**j**) and HEL (**k**,**l**) cells. Exogenous SLIT2 (200 ng ml^−1^) enhanced the inhibitory effect on colony formation. K562 and HEL cells overexpressing ROBO1 (T1160A and R886H) and ROBO2 (P1058R and P522Q) mutants displayed no significant differences in colony formation compared with the control cells. pCMV-ROBO1 M1, T1160A mutant; pCMV-ROBO1 M2, R886H mutant; pCMV-ROBO1 M3, R77H mutant; pCMV-ROBO2 M1, P1058R mutant; pCMV-ROBO2 M2, P522Q mutant; pCMV-ROBO2 M3, G107E mutant. Error bars throughout represent the s.e.m. *P<0.05, **P<0.01 and ***P<0.001 relative to the pCMV vector control group). Throughout the figure, unpaired Student's *t* tests were used to calculate all *P* values. The data shown are representative of values from three independent experiments.

Mutation screening revealed that there were no *ROBO* mutations in K562 and HEL cells, whereas a *ROBO1* mutation (p.S991N) and *ROBO2* mutation (p.I945T) were detected in U937 cells and SKM-1 cells, respectively (Supplementary Data 6). Considering the low expression level of *ROBO1* and *ROBO2* in leukaemia cell lines (Supplementary Fig. 7), we transfected the ROBO1 and ROBO2 expression vectors into K562 and HEL cell lines using the SuperFect Transfection system to observe the effect of ROBO1 or ROBO2 on the biological characteristics of leukaemia cells. After transfection, the expression of *ROBO1* and *ROBO2* increased by ∼400- to 3,500-fold, respectively (Supplementary Fig. 8). The overexpression of ROBO1 or ROBO2 induced apoptosis but did not affect the cell cycle in K562 and HEL cells (Fig. 5a–d and Supplementary Fig. 9). The overexpression of ROBO1 or ROBO2 also resulted in a decrease in cell growth and colony formation in K562 and HEL cells (Fig. 5e–g,i–l). Next, we investigated the effect of the addition of exogenous recombinant hSLIT2 together with the overexpression of ROBO1 or ROBO2 on the biological characteristics of leukaemia cells (magnification effect of SLIT2/ROBO signalling). Recent studies have reported that SLIT2, which serves as a ROBO ligand, acts as a tumour-suppressor gene in several cancers^21^,^22^,^23^. Remarkably, the addition of exogenous recombinant hSLIT2 significantly enhanced apoptosis and inhibited cell growth and colony formation in K562 and HEL cells, particularly together with the overexpression of ROBO1 or ROBO2 (Fig. 5). Taken together, our data showed that ROBO-SLIT2 signalling has a tumour-suppressive effect in MDS and leukaemia.

### ROBO1 and ROBO2 mutants impairs ROBO-SLIT2 signalling

As previously mentioned, the distribution of *ROBO* mutations was heterogeneous, and no hot spot mutations were observed (the incidence of the p.T1160A mutant of ROBO1 was relatively frequent). To further examine the specific effect of ROBO1 and ROBO2 mutations on tumorigenesis, we selected several ROBO1 and ROBO2 mutants based on targeted sequencing data (mutants from primary MDS) according to the protein prediction score, location and frequency of mutations. p.R77H, p.R886H and p.T1160A mutants in ROBO1, and p.G107E, p.P522Q and p.P1058R in ROBO2 were selected. p.R77H or p.G107E, pR886H or p.522Q and p.T1160A or P1058R were located in the extracellular immunoglobulin (Ig) region, fibronectin region and intracellular conserved cytoplasmic motif1 region, respectively. ROBO1 or ROBO2 mutants were introduced into a lentivirus vector bearing the ROBO1 or ROBO2 open reading frame and encoding an haemagglutinin (HA) tag fused at the C-terminus. The expression of the mutated protein was verified by western blot analysis after transfection (Supplementary Fig. 10). As shown in Fig. 5, in contrast to the overexpression of wild-type ROBO1, overexpression of the ROBO1 mutant (p.T1160A and p.R886H) no longer affected pro-apoptosis, inhibition of growth or colony formation in K562 and HEL cells, with a diminished receptor response of SLIT2. Similarly, overexpression of the ROBO2 (p.P1058R and p.P522Q) mutant did not produce a pro-apoptotic effect or inhibition of growth or colony formation in contrast to the overexpression of wild-type ROBO2. Nevertheless, overexpression of the ROBO1 (p.R77H) and ROBO2 (p.G107E) mutants resulted in effects that were similar to those produced by the overexpression of wild-type ROBO1 and ROBO2. These results indicated that mutations in the fibronectin and intracellular region of ROBO may significantly affect the function of ROBO and disrupt SLIT2/ROBO signalling. The blockade of ROBO-SLIT2 signalling may lead to an imbalance of cell growth and apoptosis and drive disease progression.

### Expression analysis of *ROBO1* and *ROBO2*

**Figure 6:**
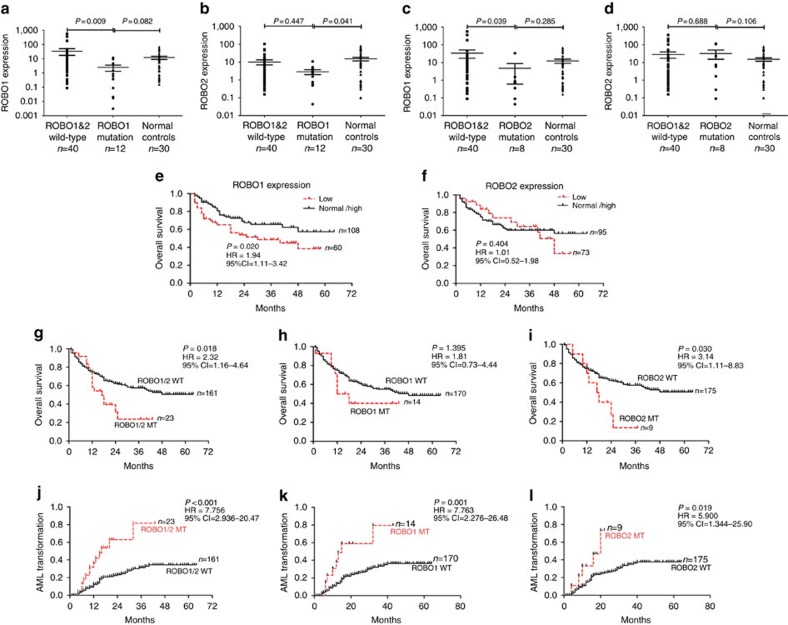
*ROBO* mutations in MDS predict poor prognosis and high AML transformation. The patients carrying the *ROBO1* mutation (*n*=12) displayed lower *ROBO1* (**a**) and *ROBO2* (**b**) expression compared with those without a *ROBO1* mutation (*n*=48; *P*=0.009, *P*=0.447). (**c**) The patients carrying the *ROBO2* mutation (*n*=8) showed lower *ROBO1* expression compared with those without a *ROBO2* mutation (*n*=52; *P*=0.039). (**d**) There was no significant difference in *ROBO2* expression between those with (*n*=8) and without (*n*=52) *ROBO2* mutations. Statistical significance was determined by one-way ANOVA's HSD test. Error bars throughout represent the s.e.m. (**e**) The patients with low *ROBO1* expression (*n*=60) displayed a shorter overall survival (OS) compared with than those with normal or high *ROBO1* expression (*n*=108). (**f**) There was no difference in OS between the patients with low *ROBO2* expression (*n*=73) and normal and high *ROBO2* expression (*n*=95). The patients with the *ROBO* mutations were significantly associated with a shorter overall survival than those without the mutation (**g**, ROBO1&2 WT versus MT, *P*=0.018; **h**, ROBO1 WT versus MT, *P*=0.395; **i**, ROBO2 WT versus MT, *P*=0.030). In addition, the patients with the *ROBO* mutations were significantly associated with high AML transformation compared with those without the *ROBO* mutations (**j**, ROBO1&2 WT versus MT, *P*<0.001; **k**, ROBO1 WT versus MT, *P*=0.001; **l**, ROBO2 WT versus MT, *P*=0.019). Statistical significance in survival analysis and AML transformation was determined by log rank test.

We also evaluated the expression of *ROBO1* and *ROBO2* mRNA in 20 patients with *ROBO* mutations, 40 MDS patients without a *ROBO* mutation and 30 non-MDS controls. The patients with a *ROBO1* mutation (*n*=12) displayed lower levels of *ROBO1* and *ROBO2* expression compared with those who did not carry a *ROBO1* mutation (one-way ANOVA's honestly significance difference (HSD) test, *P*=0.009, *P*=0.447; Fig. 6a,b). The patients with a *ROBO2* mutation (*n*=8) displayed lower levels of *ROBO1* expression than those without a *ROBO2* mutation (one way ANOVA's HSD test, *P*=0.039) (Fig. 6c). However, there were no significant differences in *ROBO2* expression between patients with and without *ROBO2* mutations (Fig. 6d). There were no significant differences in *ROBO1* or *ROBO2* between the patients with *ROBO1* or *ROBO2* mutations and the normal controls. In addition, the expression level of *ROBO1* in all MDS patients was not significantly different from that in normal controls (Supplementary Fig. 11). However, *ROBO1* expression was significantly decreased in the cases with RAEB-t in comparison to the cases with refractory cytopenia with unilineage dysplasia (RCUD) (one-way ANOVA's HSD test, *P*=0.016) and RAEB-1(one-way ANOVA's HSD test, *P*=0.011). The *ROBO2* expression level was not significantly different among the sub-groups of MDS defined by the World Health Organization classification. The expression level of *ROBO1* and *ROBO2* was not significantly different when patients were grouped by the revised International Prognostic Scoring System (IPSS-R) score.

### *ROBO1* and *ROBO2* mutations predict a poor prognosis

There were no significant differences in age, sex, diagnosis, karyotype, blood counts, bone marrow (BM) blasts or IPSS-R between the patients carrying the mutant and wild-type *ROBO* were observed (Supplementary Table 9). Nevertheless, the patients who exhibited low *ROBO1* expression displayed a shorter overall survival compared with those with normal or high *ROBO1* expression, whereas there were no differences in overall survival between the patients with low *ROBO2* expression and normal and high *ROBO2* expression (Fig. 6e,f). The patients who carried a *ROBO1* or *ROBO2* mutation (excluding two cases who had received allo-haematopoietic stem cell transplantation) were significantly associated with a shorter overall survival compared with those without a *ROBO* mutation (Fig. 6g–i). In addition, the patients with a *ROBO1* or *ROBO2* mutation were significantly associated with a high rate of AML transformation compared with those without a *ROBO* mutation (Fig. 6j–l). Furthermore, univariate and multivariate Cox analyses were conducted by integrating several risk factors including age, sex, IPSS-R, *ROBO* and 9 other mutated genes (*DNMT3A*, *BCOR*, *TP53*, *RUNX1*, *SRSF2*, *IDH*, *ASXL1*, *EZH2*, *U2AF1* and *ANRKD11*). These gene mutations are usually considered to be poor prognostic markers. Univariate analyses were performed to determine the significance of the gene mutations, and mutations in *ROBO*, *DNMT3A*, *BCOR*, *TP53*, *RUNX1* and *SRSF2* were found to be adverse prognostic factors for overall survival (Supplementary Table 10). In the multivariate analyses, *ROBO* (hazard ration (HR)=1.801; 95% confidence interval (CI)=0.977–3.322; Likelihood ratio test, *P*=0.048) and *SRSF2* (HR=1.534; 95% CI=0.612–3.203; Likelihood ratio test, *P*=0.061) appeared to be independent prognostic markers of adverse events.

## Discussion

Substantial progress has been achieved in our understanding of the pathogenesis of MDS through the characterization of somatic gene mutations, such as gene encoding transcription factors (TP53 or ETV6), epigenetic regulators involved in methylation (DNMT3A), the hydroxymethylation of cytosine (TET2, IDH1, IDH2) or the covalent modifications of histones (EZH2, ASXL1)^6^,^7^. However, these mutations are still unable to explain the mechanism underlying disease progression because of the lack of an observation of the overall process before and after progression. The factors that drive MDS to become AML remain to be elucidated.

To investigate the mechanism underlying the disease progression of MDS, we conducted, for the first time, whole-exome sequencing in three paired samples from MDS cases before and after disease progression from the lower to the higher risk stage. Among the mutated genes, we identified a recurrent somatic mutation in *ROBO1* in one patient and a related *ROBO2* gene mutation in another patient. Target sequencing of the *ROBO1* and *ROBO2* genes in 13 paired samples from progressed MDS patients further identified 4 patients with *ROBO* mutations. Together with the whole-exome sequencing, 37.5% of the *ROBO* mutations were observed in the 16 paired patients with disease progression. The *ROBO* mutations were either existed in both the lower and higher risk stages or emerged with disease progression, indicating that the *ROBO* mutation in the lower risk stage may be a fundamental driver of disease progression. Together with the results described above, deep sequencing of 209 cases revealed *ROBO* mutations in 12.4% of the patients. The percentage of *ROBO* mutations in MDS approximated that of *ASXL1* (11.4%), which has been identified as a disease-causing gene in MDS^10^,^11^,^12^. The mutations that were commonly observed together with *ROBO* mutations included *DNMT3A*, *SF3B1*, *ANKRD11* and *ASXL1*. Two cases revealed that both *ROBO* mutations and *SF3B1* had a relatively prolonged survival because of the classification as refractory anemia with ring sideroblast or RCMD-RS. All of the cases carrying a *ROBO* mutation and *DNMT3A*, *ANKRD11* or *ASXL1* mutations had a poor prognosis with a high rate of AML transformation. Mutations in *DNMT3A*, *ANKRD11* and *ASXL1* are considered to predict poor survival in MDS as well as in other cancers^6^,^7^,^18^,^24^. Thus, these gene mutations together with a *ROBO* mutation may further facilitate disease progression. In addition to our report, a recent study investigating multiple myeloma indicated that *ROBO1* mutations were frequent and may affect disease prognosis.

In the present study, in addition to *ROBO* mutations, the frequent incidence of CN loss and LOH of *ROBO1* and *ROBO2* were also observed in patients with disease progression. Notably, the patients who carried a *ROBO* mutation commonly harboured CN loss and LOH at the *ROBO1* and *ROBO2* locus. Gene expression analysis verified a low expression level of *ROBO1* and *ROBO2* in MDS, particularly in patients carrying *ROBO* mutation. Previous reports have demonstrated that *ROBO1* or *ROBO2* expression is frequently lost in many cancers and is associated with a loss of heterozygosity of these genes^25^,^26^,^27^,^28^,^29^, which is consistent with our findings. Together with the data from the mutation analysis, it can be speculated that the abnormalities in protein coding because of mutation or deficiencies in gene expression by CN loss or LOH contribute to the impairment of ROBO function. This mode of gene inactivation is analogous to the ‘two-hit' theory^30^, in which tumour-suppressor genes are inactivated by a biallelic mutation (homozygous, ∼100%). However, monoallelic mutations (heterozygous, ∼50%) are more common in leukaemia or MDS. A monoallelic mutation together with CN loss in the other allele with *ROBO1* or *ROBO2* constructs is similar to the ‘two-hit' possibility in MDS that may drive disease progression. A similar model with co-occurrences of mutation and loss of expression of *ROBO1* and *ROBO2* has been reported in colorectal cancers, supporting our speculation^31^.

Regarding the function of ROBO, it has been reported that ROBO receptors bind to the SLIT2 protein and play a critical role in the development of axon guidance^32^. In addition to axon guidance, the activation of ROBO-SLIT2 signalling clearly promotes cell apoptosis and cycle blockade in the G0 and G1 phase and inhibits cell proliferation and migration in several solid cancers^21^,^22^,^23^. Thus, *ROBO1* and *ROBO2* may be considered to be tumour-suppressor genes in cancers, a notion that is also supported by our SNV and SNP data in lower and higher risk cases with MDS. To further confirm the anti-tumour effect of ROBO1 and ROBO2 in MDS and leukaemia cells, we further performed experimetns *in vitro* by overexpressing of wild-type and mutant ROBO in leukaemia cells. The results showed that ectopic expression of ROBO1 or ROBO2 produced varying degrees of an anti-tumour effect in leukaemia cells, whereas extracellular ROBO mutants lost this effect. In addition, the extracellular ROBO mutants also severely impaired the interaction of ROBO-SLIT2, leading to the inactivation of ROBO-SLIT2 signalling. Our results revealed that *ROBO1* and *ROBO2* should be considered as tumour-suppressor genes in MDS and AML. The mechanism underlying ROBO-SLIT2 signalling involved in the pathogenesis of MDS remains unknown. Several previous studies have revealed that ROBO-SLIT2 signalling can inactivate the AKT/GSK3/β-catenin pathway in solid cancers^22^,^33^,^34^. It is well-known that the AKT/GSK3/β-catenin pathway is over-activated in MDS especially high-grade MDS, and it is associated with elevated cell proliferation and resistance to apoptosis^35^,^36^. *ROBO* mutations lead to the inactivation of ROBO-SLIT2 signalling, and relieved the depressive effect on the AKT/GSK3/β-catenin pathway, which can result in uncontrolled cell proliferation and thus disease progression in MDS. Further studies should be conducted to identify whether other pathways are affected by ROBO-SLIT2 signalling act through. Based on the present findings, we proposed that *ROBO1* and *ROBO2* should be considered as tumour-suppressor genes in MDS and AML. The characteristics of *ROBO1* and *ROBO2* as tumour-suppressor genes were also observed in the clinical results. The patients with *ROBO* mutations or low expression levels of *ROBO1* or *ROBO2* displayed shorter survival and higher AML transformation compared with those without mutations. *ROBO* mutations were defined as an independent prognostic factor, further supporting the importance of alterations of *ROBO1* and *ROBO2* as drivers of MDS progression.

In summary, this is the first report of whole-exome sequencing of paired samples from MDS cases with disease progression and the first description of recurrent, validated *ROBO1* and *ROBO2* mutations in MDS that confer a worse clinical outcome. *In vitro* studies has shown that *ROBO1* and *ROBO2* function as tumour-suppressor genes. These tumour-suppressive effects could be weakened by mutation, CN loss or LOH. Our results provide new insight for future mechanistic studies and targeted therapy for MDS.

## Methods

### Patients and samples

The study population consisted of 225 samples from 209 patients with MDS at Shanghai Jiao Tong University Affiliated Sixth People's Hospital from 2006 to 2014. MDS was diagnosed in accordance with the minimum diagnostic criteria (Vienna, 2006)^37^. The classification and prognostic risk scoring of MDS were performed according to the World Health Organization criteria^38^ and the IPSS-R^39^. Six samples from three paired patients at the time of diagnosis (RA, RN or RCMD) and disease progression stage (RAEB-2) were obtained for whole-exome sequencing. Twenty-six samples from 13 paired patients at the diagnosis (lower risk MDS) and the disease progression stage (higher risk MDS), as well as the remaining 193 samples at the visit, were obtained for targeted deep sequencing. BM samples were obtained by aspiration, and mononuclear cells were collected by density gradient centrifugation. Oral mucosal epithelial samples were collected as germline controls. All of the subjects provided written informed consent for genetic analysis under a protocol that was approved by the Ethics Committee of Shanghai Jiao Tong University Affiliated Sixth People's Hospital.

### GDNA preparation and whole-exome sequencing

GDNA was extracted from bone marrow mononuclear cells, and matched oral mucosa epithelium was extracted from the MDS individuals. The purity (optical density (OD)_260/280_>1.8) and concentration (50 ng μl^−1^) of the gDNA met the sequencing requirements. The gDNA library was prepared using a TruSeq DNA Sample Preparation Kit (Illumina) in accordance with the manufacturer's protocols. In-solution exome enrichment was performed using a TruSeq Exome Enrichment kit (Illumina) according to the manufacturer's instructions. The enriched DNA samples were sequenced via 2 × 100 paired-end sequencing using a Hiseq2000 Sequencing System (Illumina). Illumina Sequencing Control v2.8, Illumina Off-Line Basecaller v1.8 and Illumina Consensus Assessment of Sequence and Variation v1.8 software (Illumina) were used to produce 100-base pair (bp) sequence reads.

### Sequencing data processing and mutation calling

Data processing was divided into two steps: (i) generation of a binary sequence alignment map (BAM) file (using SAMtools) for paired BM and mucosal epithelial samples for each case and (ii) detection of somatic SNVs and indels by comparing the BM and mucosa epithelial BAM files. The sequence reads were aligned to the human reference genome (hg19) using the Burrows-Wheeler Aligner^40^ with default parameters. Variants were identified using the Genome Analysis Toolkit^41^ and VarScan software^42^. Coverage analysis was determined using the Picard software CalculateHsMetrics tool. Reads that matched exonic regions, including exon–intron boundaries, were analysed. SNVs and indel analyses were performed using different filtering steps. Relevant mutations in all of the genes were subsequently prioritized manually.

### Targeted gene sequencing

Twenty-six marker genes were screened for the detection of mutations in 206 patients (excluding the three paired patients undergoing whole-exome sequencing) by sequencing using the MiSeq Benchtop Sequencer (Illumina). These genes were selected according to the following criteria: (i) genes that were recurrently mutated in the three paired patients; (ii) genes that were newly presented in the disease progression stage; (iii) genes that were identified as first priority in the calling quality and (iv) genes that were related to the development of cancer. To identify the mutations in the highlighted genes, we designed PCR primers using the primerXL pipeline. Three hundred and eighty oligonucleotide pairs were produced and encompassed all of the CDSs and most of the untranslated regions of the 26 genes. The amplification reactions were conducted using an AB 2720 Thermal Cycler (Life Technologies Corporation) with the following cycling conditions: 95 °C for 2 min; 11 cycles of 94 °C for 20 s, 63 °C per cycle for 40 s, 72 °C for 1 min; 24 cycles of 94 °C for 20 s, 65 °C for 30 s, 72 °C for 1 min and 72 °C for 2 min. The PCR products were used generate a library for further detection, and the DNA-adapter-ligated and -indexed fragments from ten libraries were then pooled and hybridized. After hybridization of the sequencing primer, base incorporation was performed using the MiSeq Benchtop Sequencer in a single lane following the manufacturer's standard cluster generation and sequencing protocols for 250 cycles of sequencing per read to generate paired-end reads, including 250 bp at each end and 8 bp of the index tag.

### Detection of CN variation and LOH

The DNA from MDS was prepared for hybridization to the Affymetrix CytoScan 750 K array (750,000 probes) according to the manufacturer's protocol. A total of 250 ng of isolated DNA per sample was digested with *Nsp*I, and the sample was subsequently ligated, PCR-amplified and purified, fragmented, biotin-labelled and hybridized for use in a CytoScan 750 K Array (Affymetrix). The data were analysed using the Nexus Copy Number (version 7.5; Biodiscovery Inc.) software programme, and they were normalized using the SNP-FASST2 segmentation algorithm. The normalized probe intensity and allele ratio data were visualized in Nexus v7.5. In addition to the microarray analysis, the TaqMan Copy Number Assay was also used to quantitatively analyse the CN of *ROBO1* and *ROBO2*. The primers and probes were purchased from Applied Biosystems Inc, and the assay was performed according to the manufacturer's instructions. Each replicate was normalized to RPP14 (a reference gene) to obtain a ΔCt (FAM dye Ct- VIC dye Ct), and an average ΔCt for each sample was calculated. All of the samples were normalized to a calibrator sample to determine the ΔΔCt. The relative quantity was 2-ΔΔCt, and the copy number was 2 × relative quantity.

### Cell culture and reagents

Leukaemia cell lines, including K562, HEL and U937, were obtained from American Type Culture Collection. The MDS-derived AML cell line SKM-1 was a gift from Professor Nakagawa^43^. The cell lines were maintained in complete medium (RPMI 1640 supplemented with 10% heat-inactivated fetal bovine serum, 1% glutamine and 1% sodium pyruvate). All the cell lines are not MDS cells. Recombinant human SLIT2-N (purchased from PeproTech Inc.) was dissolved in medium to a working concentration of 100 ng ml^−1^.

### Transfection of mutant or wild-type ROBO1 and ROBO2

*ROBO1* or *ROBO2* mutations were introduced into the GV167 (CMV-HA-MCS-Neomycin) vector bearing the *ROBO1* or *ROBO2* open reading frame and encoding a C-terminal fusion of the HA tag using a Fast Mutagenesis system kit (Jikai Biotech). The obtained constructs were verified using Sanger sequencing and transfected into the leukaemia cell lines using the SuperFect Transfection system (Qiagen) according to the manufacturer's instructions. Transgene expression was verified by real-time PCR and protein blot analysis for ROBO1 or ROBO2.

### RNA preparation and quantitative PCR

The total RNA was extracted using the RNeasy system (Qiagen) following the manufacturer's instructions, and the RNA was reversed transcribed into cDNA. The PCR reactions were performed using the ABI PRISM 7500 System (Applied Biosystems) and SYBR Green Master Mix (Takara). The relative gene expression was calculated using 2^-ΔΔCt^. The detailed sequences of the primers can be acquired from PrimerBank constructed by Harvard University^44^.

### WST-1 proliferation assay

The transfected cells were plated in 96-well plates at a density of 5 × 10^3^ cells per well in triplicate. After culture, ten microliters of WST-1 working solution (Keygen, Nanjing, China) was added to each well, and the cells were incubated for 2 h. The absorbance at 450 nm was measured using a microplate reader. The rate of relative cell growth was calculated as follows: relative growth rate=each time point (OD^transfected well^−OD^blank well^)/(OD^control well^−OD^blank well^).

### Colony formation assay

Transfected cells were plated in six-well plates with Methocult H4434 methylcellulose medium containing stem cell factor (SCF), granulocyte-macrophage colony-stimulating factor (GM-CSF), interleukin 3 (IL-3) and erythropoietin (R&D Technologies) at 500 cells per well in triplicate wells per condition. Fourteen days after incubation in a humidified incubator at 37 °C, colonies containing at least 30 cells were counted.

### Cell cycle analysis

A total of 5 × 10^4^ transfected cells were washed with cold PBS, fixed in 70% ethanol, washed again with PBS and then re-suspended in 1 ml of propidium iodide staining reagent (50 mg ml^−1^ propidium iodide and 1 mg ml^−1^ RNAse). The samples were incubated in the dark for 30 min before cell cycle analysis. The cell cycle distribution was measured using a FACS Calibur instrument. The percentages of cells in G1, S and G2 phases were calculated using Cellquest software.

### Western blotting analysis

K562 and HEL cells (10^7^) were lysed with cell lysis buffer. Total cellular extracts were fractionated in 10% SDS–polyacrylamide gels, electroblotted onto polyvinylidene difluoride membranes (Millipore) and reacted with primary antibodies including anti-ROBO1 (1:200), anti-ROBO2 (1:500), anti-HA (1:500) and GAPDH (1:1,000). Secondary goat anti-mouse antibodies (1:1,000) conjugated to horseradish peroxidase were used for enhanced chemoluminescence (Pierce Chemical), and the membranes were exposed to film. Immunoreactivity was determined using the enhanced chemiluminescence method (Pierce Chemical). Anti-ROBO1 (catalogue number MAB7118), anti-ROBO2 (catalogue number MAB3147), anti-GAPDH (catalogue number MAB5718) antibodies and goat anti-mouse IgG HRP-conjugated antibody (catalogue number HAF007) were purchased from R&D System Inc. Anti-HA (catalogue number AB9110) antibody and goat anti-rabbit IgG HRP-conjugated antibody (catalogue number AB97051) were purchased from Abcam Inc.

### Statistical analysis

Statistical analyses were conducted using SPSS software version 18.0. The association of mutations with clinical characteristics was analysed by the *χ*^2^ test. The Kaplan–Meier test was used for univariate survival analysis. Multivariate Cox proportional hazards models were used to calculate the hazard ratios and 95% confidence intervals of the associations between the risk factors and survival. The comparison of two independent samples was assessed using the two-tailed Student's *t*-test (normal distribution) or non-parametric Mann–Whitney *U*-test (non-normal distribution). Multiple pairwise comparisons were performed using a one-way ANOVA. *P* value less than 0.05 was considered statistically significant.

## Additional information

**Accession codes:** Whole-exome sequencing data have been deposited in the National Center for Biotechnology Information Sequence Read Archive under accession number SRP062250. SNP array data have been deposited in the NCBI Gene Expression Omnibus under GEO accession number GSE73005.

**How to cite this article:** Xu, F. *et al*. Whole-exome and targeted sequencing identify *ROBO1* and *ROBO2* mutations as progression-related drivers in myelodysplastic syndromes. *Nat. Commun.* 6:8806 doi: 10.1038/ncomms9806 (2015).

## Supplementary Material

Supplementary Information Supplementary Figures 1-11 and Supplementary Tables 1-10.

Supplementary Data 1 Single-nucleotide variants (SNV) indentified by whole-exome sequencing in the three paired MDS patients.

Supplementary Data 2 Screened SNVs in the three paired MDS patients through whole-exome sequencing.

Supplementary Data 3 Targeted sequencing information in additional 13 paired patients

Supplementary Data 4 Targeted sequencing information in 26 MDS patients with ROBO mutations

Supplementary Data 5 Snapshot sequencing information in normal older adults and MDS patients with ROBO mutations

Supplementary Data 6 Targeted sequencing information in leukemia and lynmphoma cell lines
