# Supplementary material for: Whole-exome and targeted sequencing identify ROBO1 and ROBO2 mutations as progression-related drivers in myelodysplastic syndromes
Source: Nat Commun. 2015 Nov 26;6:8806. doi: 10.1038/ncomms9806 (PMC4674765; doi:10.1038/ncomms9806)
Supplement: Supplementary Information — Supplementary Figures 1-11 and Supplementary Tables 1-10. [file ncomms9806-s1.pdf]

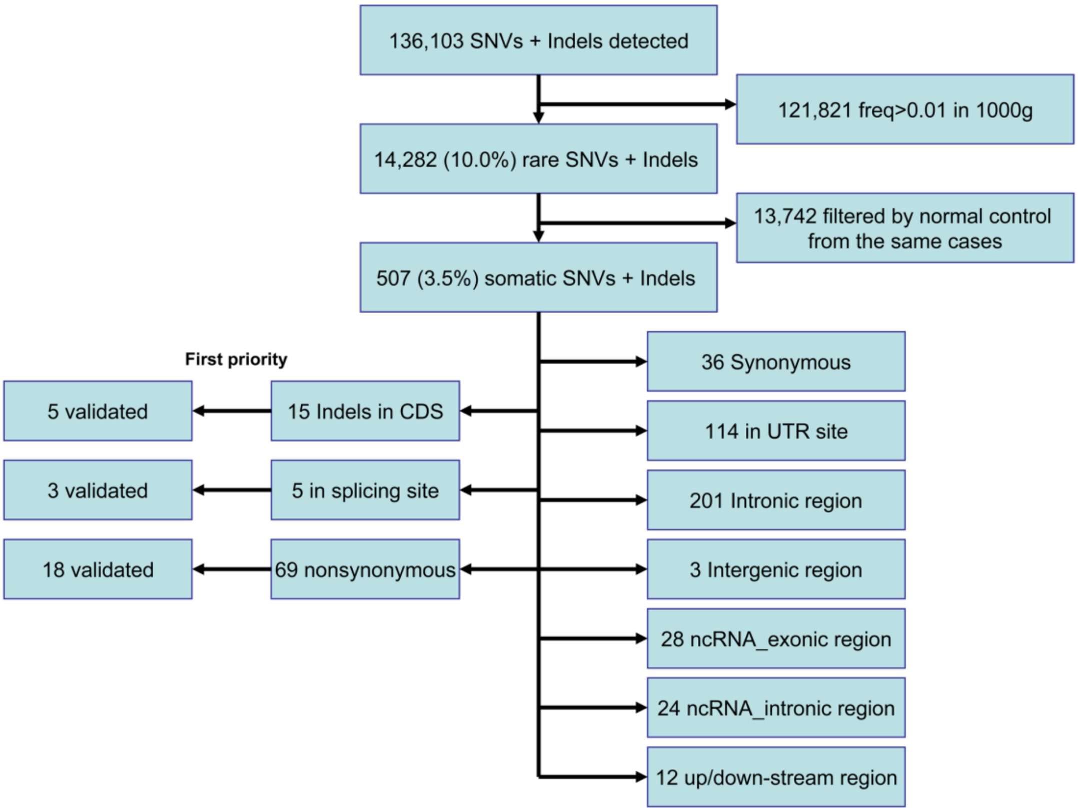

Supplementary Figure 1 Flow chart for the identification of single-nucleotide variants (SNVs) in the sequencing database of three paired patients. 1000g, 1000 Genomes database; indels, small insertions and deletions; CDS, coding sequence; UTR, untranslated regions; ncRNA, non-coding RNA.

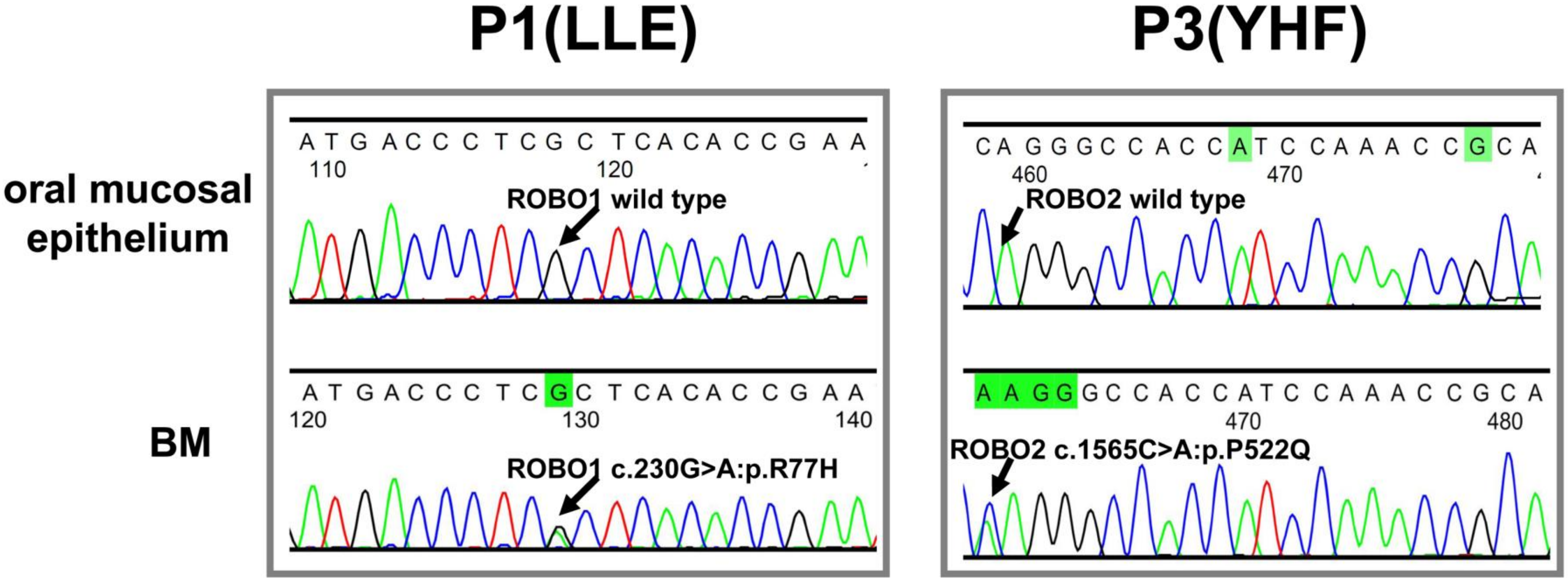

Supplementary Figure 2 Sanger sequencing of the oral mucosal epithelium and bone marrow mononuclear cells confirmed that *ROBO*1 and *ROBO*2 mutations were somatic changes in two paired patients (P1 and P3)**.**

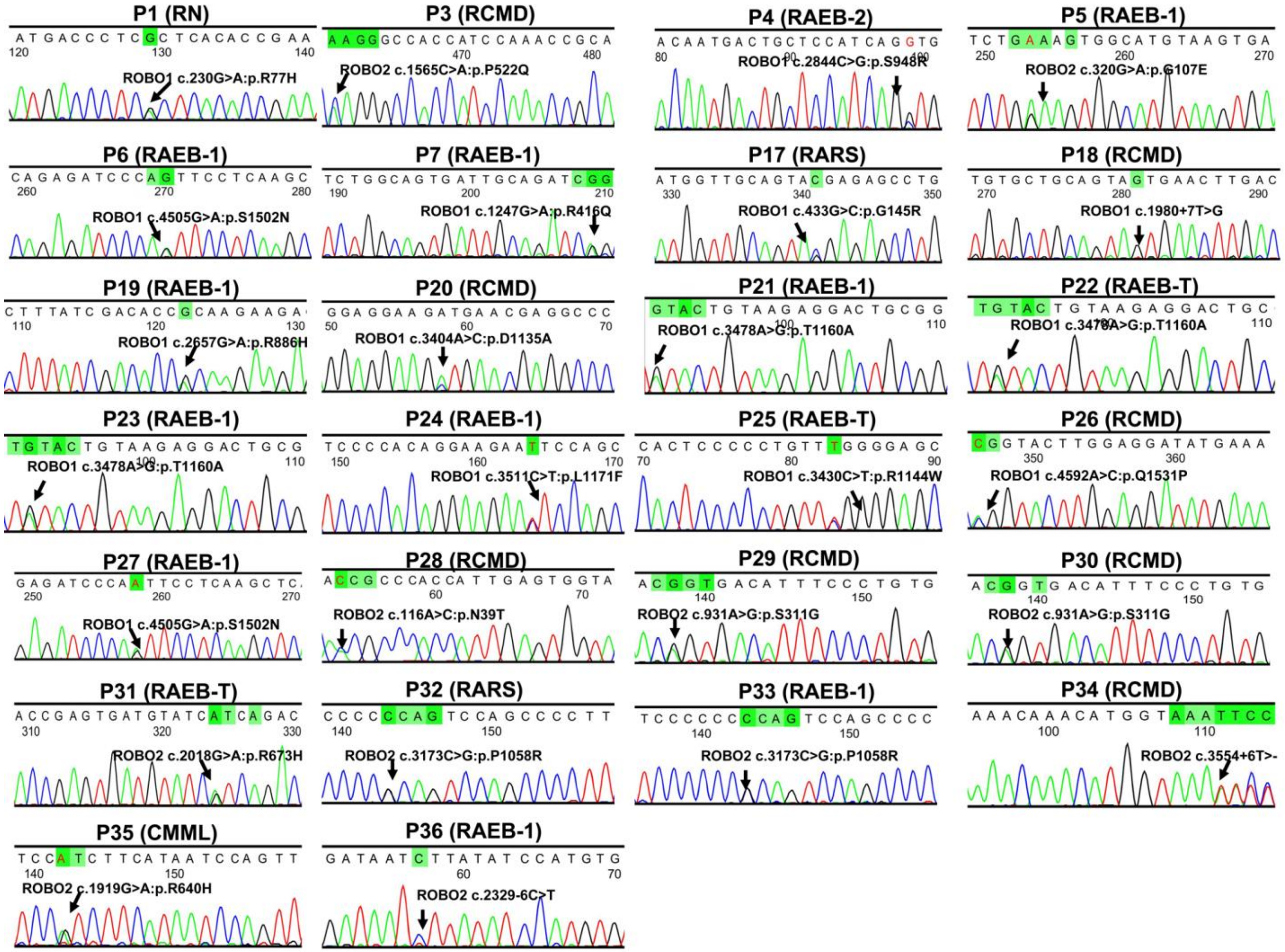

Supplementary Figure 3 Results of Sanger sequencing in 26 patients with *ROBO1* or *ROBO2* mutations.

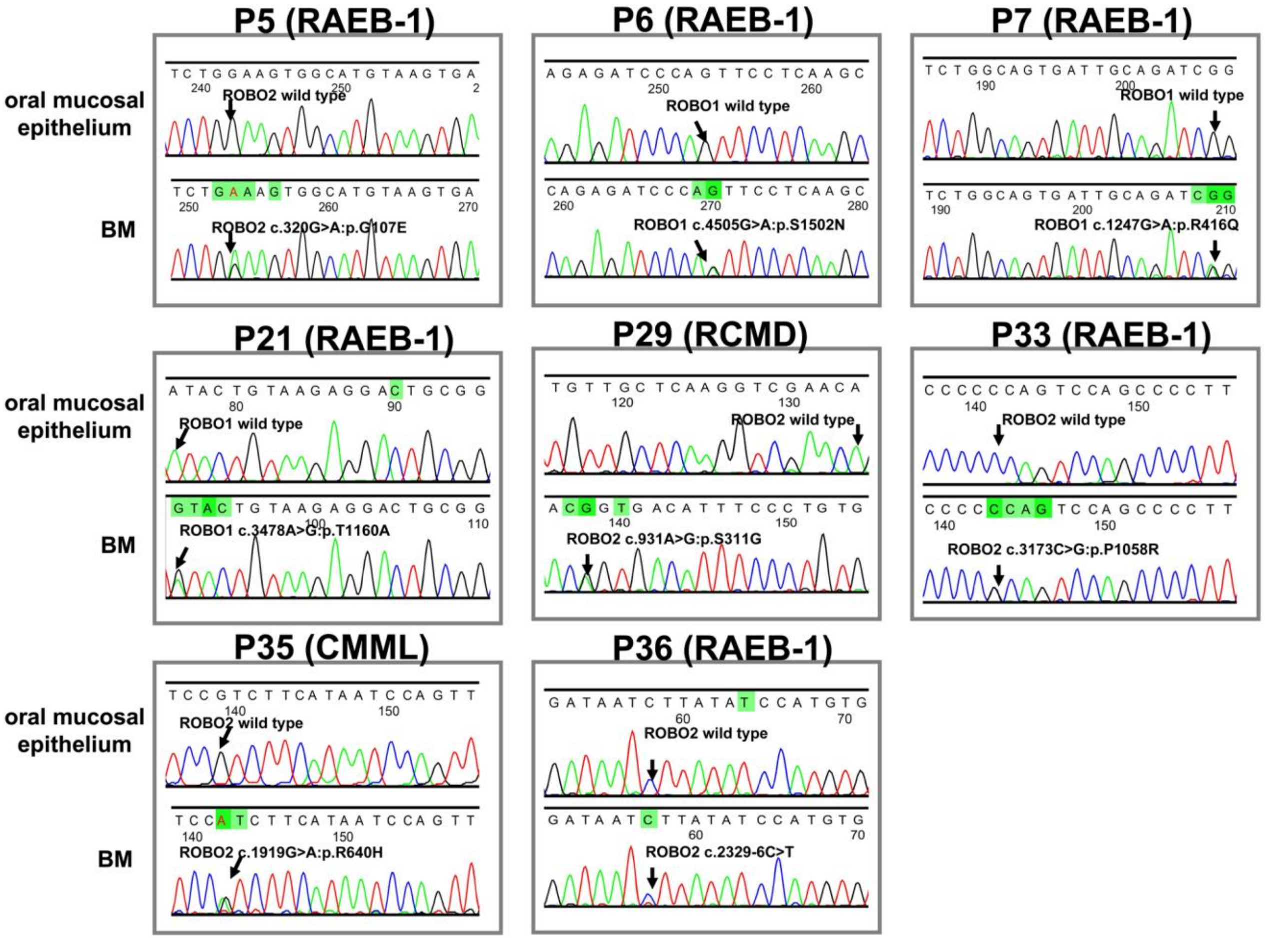

Supplementary Figure 4. Sanger sequencing in the oral mucosal epithelium and bone marrow mononuclear cells of 8 patients with *ROBO1* or *ROBO2* mutations.

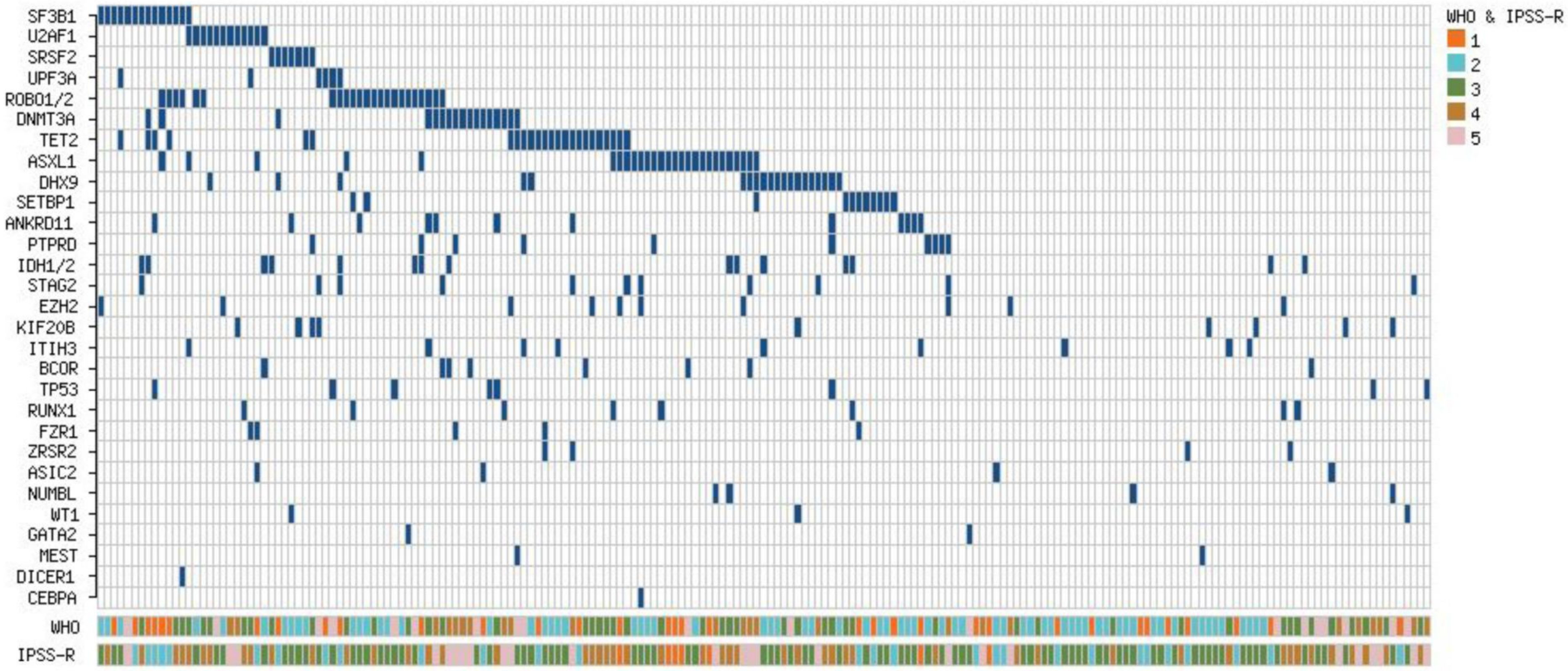

Supplementary Figure 5. Distribution of gene mutations in 195 MDS patients. Mutations are colored in the blue grid. The bottom bars show WHO classification (1, RCUD/RARS; 2, RCMD/RCMD-RS; 3, RAEB-1/CMML-1; 4, RAEB-2/CMML-2; 5, RAEB-T) and IPSS-R (1, very low; 2, low; 3, int; 4, high; 5, very high). RCUD, refractory cytopenia with unilineage dysplasia; RARS, refractory anemia with ringed sideroblasts; RCMD, refractory cytopenia with multilineage dysplasia; RAEB-1, refractory anemia with excess blasts-1; RAEB-2, refractory anemia with excess blasts-2. IPSS-R, revised international prognostic scoring system.

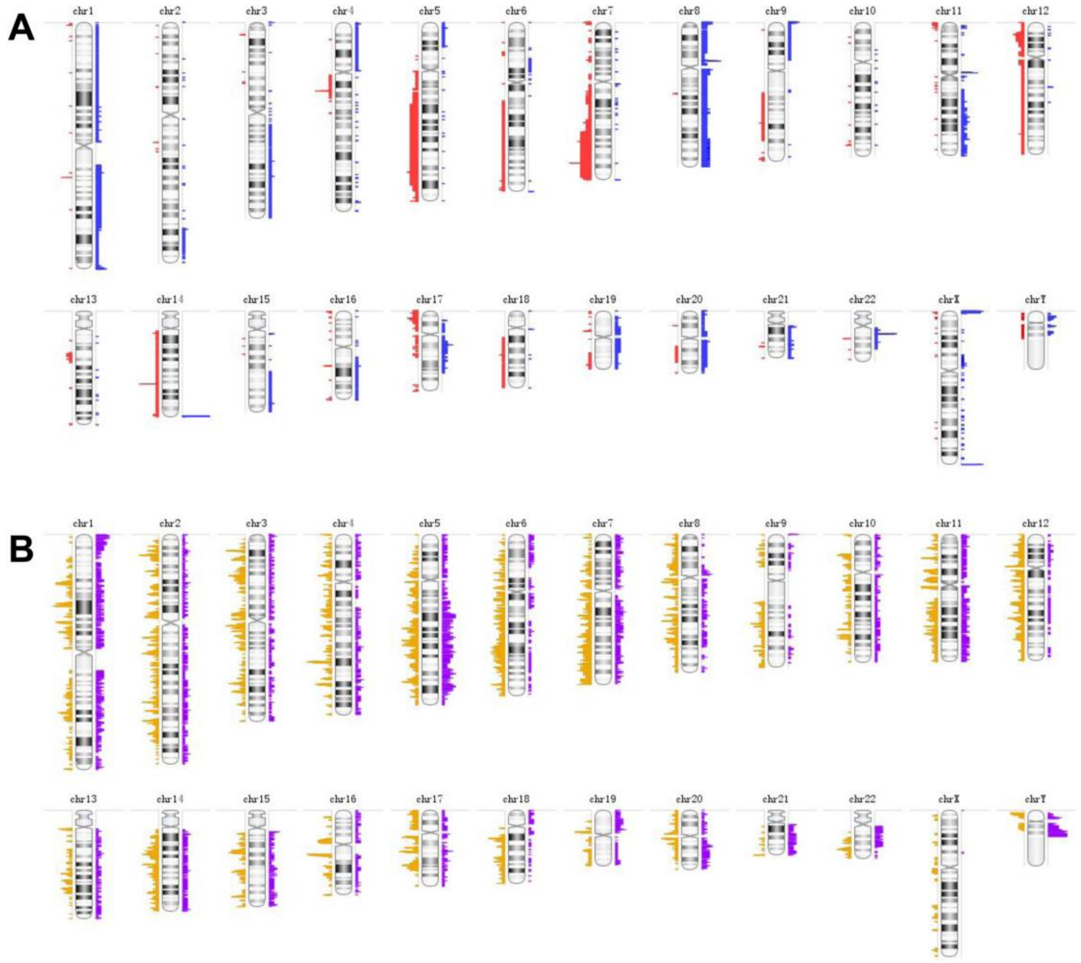

Supplementary Figure 6. Distribution of somatic copy number variations (CNVs) and loss of heterogeneity (LOH) in MDS patients. (**A**) CN loss and gain are indicated by the red and blue lines, respectively; (**B**) LOH and allelic imbalance are shown in yellow and purple waves, respectively.

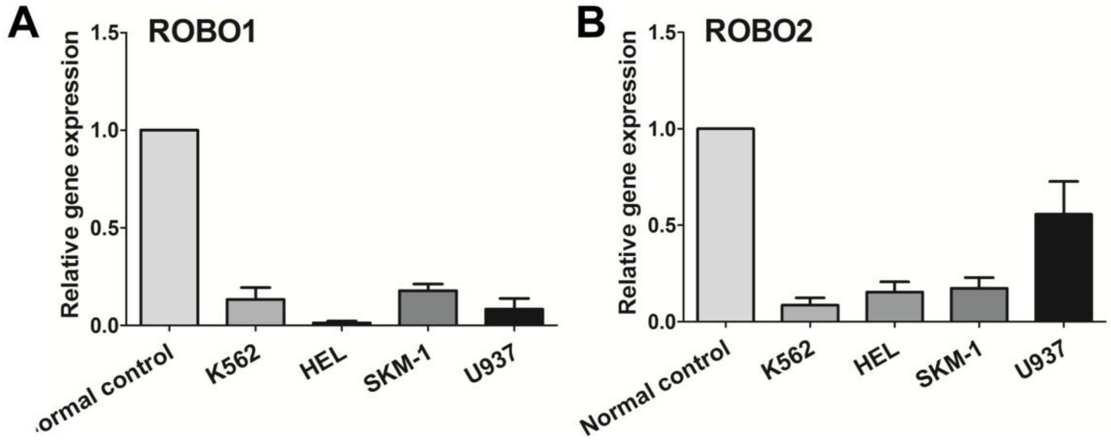

Supplementary Figure 7. The expression of *ROBO1* and *ROBO2* in normal controls and leukemia cells. K562, HEL and SKM-1 cells exhibited low expression levels of *ROBO*1 (**A**) and *ROBO2* (**B**). Error bars throughout represent the s.e.m. The detection of ROBO1 or ROBO2 mRNA by quantitative RT-PCR was replicated three times.

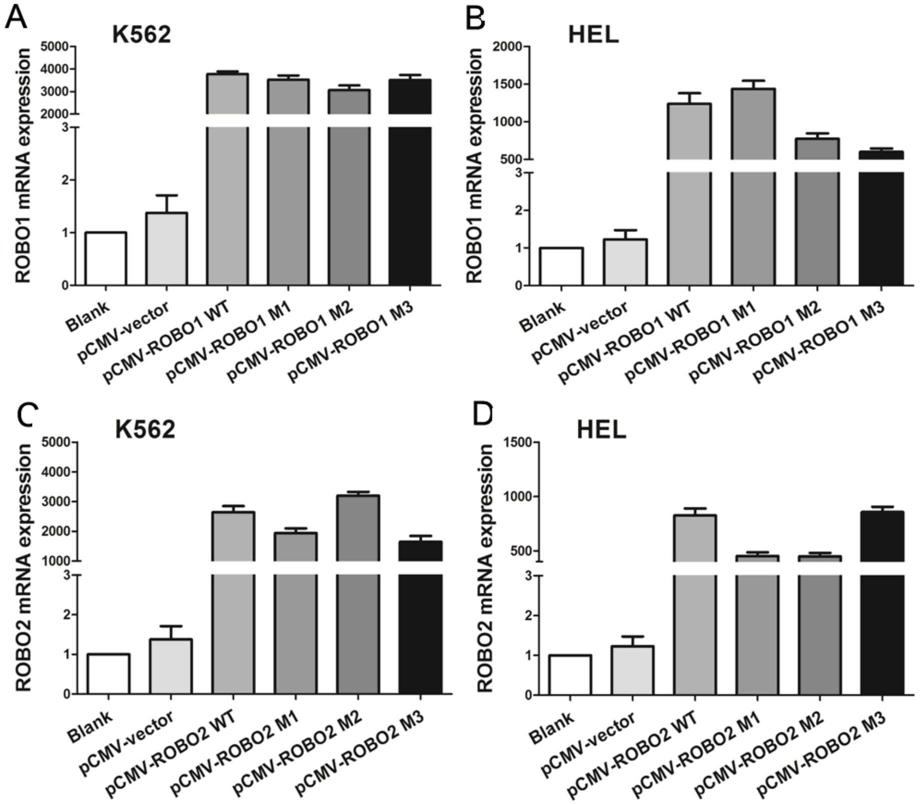

Supplementary Figure 8. Evaluation of the transfection efficiency in leukemia cells by quantitative RT-PCR. (A-D) K562 and HEL leukemia cells were, respectively, transfected with the *ROBO*1/2 wild type or mutant, and the mRNA expression of both genes was determined by real-time quantitative PCR after 48 hours. GAPDH served as an internal control. The fold change in transfected cells was relative to that in blank cells. Error bars throughout represent the s.e.m. The detection of ROBO1 or ROBO2 mRNA by quantitative RT-PCR was replicated three times.

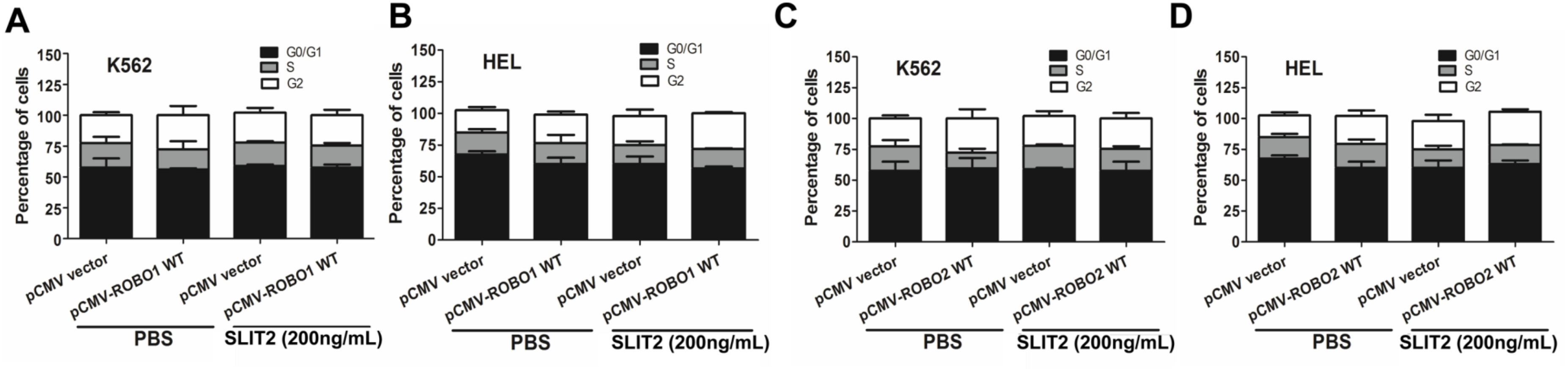

Supplementary Figure 9. Analysis of the cell cycle after transfection of ROBO1 or 2 wild type or mutant. K562 (**A and C**) and HEL (**B and D**) leukemia cells were, respectively, transfected with ROBO1 or 2 wild type or mutant, and the cell cycle was determined by flow cytometry after 48 hours. There were no differences observed between ROBO1 or 2 over-expressed cells and control cells, even during co-culture with SLIT2. Error bars throughout represent the s.e.m. The determination for cell cycle was replicated three times.

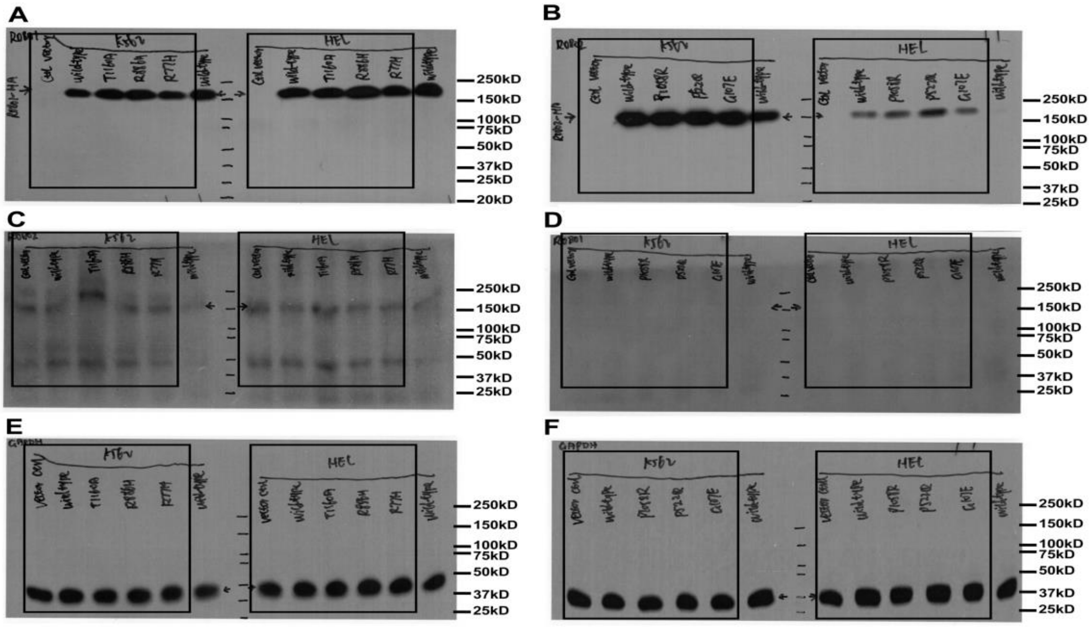

Supplementary Figure 10. Expression of the mutated protein was verified by western blot after transfection. K562 and HEL cells were transfected with vector expressing wild-type or mutant ROBO1, or wild-type or mutant ROBO2. Anti-HA tag antibody was used to assess the expression of ROBO1 (**A**) and ROBO2 (**B**). In addition, the background expression of ROBO2 (**C**) and ROBO1 (**D**) and the GAPDH (**E** and **F**) as internal reference was also determined by protein blotting analysis. The western blot experiments were replicated three times.

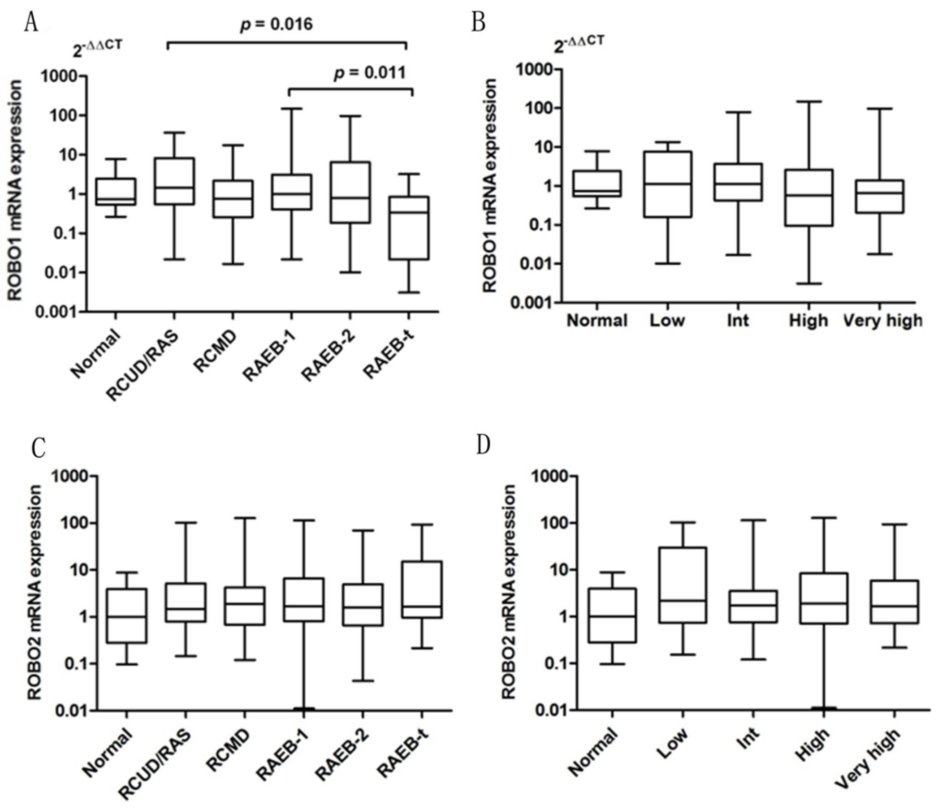

Supplementary Figure 11. Expression analysis of *ROBO*1/2 in MDS subtypes. (**A**)The expression level of *ROBO*1 mRNA in all MDS patients was not significantly different from that in normal controls. However, *ROBO*1 expression was significantly decreased in cases with RAEB-t when compared with cases with RCUD (*P*=0.016) or RAEB-1(*P*=0.011). (**B**) The expression level of *ROBO*1 was not significantly different among the groups according to the IPSS-R score. (**C**) The expression level of *ROBO*2 was not significantly different among the sub-groups of MDS defined by the WHO classification. (**D**) There were no differences in *ROBO*2 expression in the different groups according to the IPSS-R score. Error bars throughout represent the s.d. Statistical significance was determined by one way ANOVA HSD test.

Supplementary Table 1 Clinical characteristics of three paired MDS cases before and after disease progression

Patient ID	Gender	Age	WHO subtype		Blast (%) in bone marrow		Karyotype		IPSS-R		Progression time
			Before	After	Before	After	Before	After	Before	After	
P1 (LLE)	F	68	RN	RAEB-2	4.5	14.2	Normal	Normal	2.5	6	28 months
P2 (LZL)	M	70	RCMD	RAEB-2	3.0	14.1	Normal	Normal	3.5	5.5	3 months
P3 (YHF)	M	54	RA	RAEB-2	3.0	12.6	Normal	Normal	2	5.5	4 months

P1 toP2 had both bone marrow (BM) samples collected at the primary diagnosis and matched oral mucosal epithelial samples, which were defined as the whole-exome sequencing set. IPSS-R, revised international prognostic scoring system. RN, refractory neutropenias; RA, refractory anemia; RCMD, refractory cytopenia with multilineage dysplasia; RAEB-2, refractory anemia with excess blasts-2. P1/2/3, Patient 1/2/3.

Supplementary Table 2 Sequencing depth and coverage of the nine paired initial sequencing samples

	P3-low	P3-high	P3-ctrl	P2-low	P2-high	P2-ctrl	P1-low	P1-high	P1-ctrl
**ReadsNum (M)**	108.76	88.51	83.04	67.12	109.09	58.56	57.1	47.96	44.19
**Base(G)**	10.98	8.94	8.39	6.78	11.02	5.91	5.77	4.84	4.46
**Q20**	93.70%	94.60%	95.00%	86.56%	84.27%	87.03%	86.79%	86.19%	87.08%
**Q30**	86.09%	87.68%	88.12%	74.22%	71.22%	74.83%	74.56%	74.04%	74.93%
**ON BAIT BASES (M)**	4322.9	3620.7	3485.8	3272.6	5111.4	2774.2	2746.5	2156.2	2076.5
**MEAN_TARGET_COVERAGE**	69.97	58.67	56.44	52.98	82.75	44.94	44.54	34.95	33.68
**PCT_TARGET_BASES_2X**	95.26%	94.74%	94.23%	92.38%	92.71%	90.06%	88.99%	92.82%	90.20%
**PCT_TARGET_BASES_10X**	88.23%	89.06%	87.61%	83.67%	81.22%	77.24%	75.55%	81.55%	75.67%
**PCT_TARGET_BASES_20X**	77.12%	79.16%	77.61%	71.74%	67.04%	63.68%	62.06%	63.83%	58.25%
**PCT_TARGET_BASES_30X**	65.74%	66.90%	65.80%	59.84%	56.07%	51.99%	50.72%	46.28%	43.41%

Supplementary Table 3 Summary of SNVs in three paired patients.

Variant	P1 (LLE)		P2 (LZL)		P3 (YHF)	
	before	after	before	after	before	after
**All SNVs detected**	125	158	175	189	232	267
**Coding Region**						
Missense	15	15	14	18	49	62
Nonsense	0	0	1	0	2	2
Synonymous	8	16	8	11	6	6
indel	3	3	8	8	11	11
**Noncoding, transcribed**						
5' UTR	6	7	5	6	14	20
3'UTR	41	55	60	58	44	47
Splice site	1	2	1	0	3	3
ncRNA_exonic region	5	8	9	9	11	13
ncRNA_intronic region	7	9	2	3	8	12
Intronic	35	38	64	72	79	85
Intergenic	1	1	1	1	1	1
Up/down-stream region	3	4	2	3	4	5

indels, small insertions and deletions; UTR, untranslated regions; ncRNA, non-coding RNA.

Supplementary Table 4 Somatic mutations in 26 genes in three paired patients

Annotated	Mutation	Position	Allele Change	Amino Acid Change	RefSeq	Polyphen Prediction	Sample Number
ANKRD11	missense	chr16: 89351826	G>A	p.T375M	NM_013275	probably damaging	P1-high
ASIC2	missense	chr17: 31439018	G>A	p.T259M	NM_183377	damaging	P2-low and high
ASXL1	frameshift deletion; frameshift insertion	chr20: 31022403-31022425; chr20: 31022440-31022440	*/- CACCAC TGCCATA GAGAGGCGGC; */+A	p.630_637del; p.G642fs	NM_015338	damaging	P1-high, P2-low
DACH1	nonframeshift insertion	chr13: 72440443-72440443	*/+ CTGCTG	p.S155delinsSSS	NM_004392	benign	P3-low and high
DHX9	missense	chr1:182850508	T>C	p.F912L	NM_001357	probably damaging	P3-low and high
FZR1	missense	chr19:36394707	C>T	p.P453S	NM_016263	damaging	P2-low and high
GTF2B	missense	chr1: 89325822	C>T	c.405+1G>A	NM_001514	unknown	P2- high
HCST	missense	chr19: 36394707	C>A	p.P48Q	NM_014266	damaging	P2-low and high
ITIH3	missense	chr3: 52831917	G>A	p.A212T	NM_002217	probably damaging	P1-high
KIF20B	splicing	chr10:91518462	G>T	c.4384-1G>T	NM_016195	unknown	P2-high
MED23	missense	chr6:131924189	G>T	p.Q644K	NM_015979	probably damaging	P1-high
MEST	missense	chr7:130138092	T>C	p.I142T	NM_001253902	benign	P3-low and high
NUMBL	missense	chr19: 41173662	T>A	p.Q514L	NM_004756	damaging	P2-low and high
PHF14	missense	chr7: 11076087	G>C	p.E549Q	NM_014660	probably damaging	P3-low and high

PTPRD	missense	chr9:8484351	C>T	p.E645K	NM_130393	probably damaging	P1-low and high
RBM10	missense	chrX: 47040937-47040938	*/-CT	p.412_413del	NM_001204466	unknown	P2-low and high
ROBO1	missense	chr3:78987903	C>T	p.R77H	NM_001145845	damaging	P1-low and high
ROBO2	missense	chr3: 77612363	C>A	p.P522Q	NM_002942	damaging	P3-low and high
SRSF2	missense	chr17:74732959	G>C	p.P95R	NM_003016	probably damaging	P1-low and high
ST8SIA1	splicing	chr12: 22487648-22487648	*/+CA	-	-	-	P2-low and high
TDG	missense	chr12:104379486	G>A	p.G357D	NM_003211	damaging	P1-low and high
U2AF1	missense	chr21: 44524456	G>A	p.S34F	NM_006758	damaging	P2-low and high
UPF3A	frameshift deletion	chr13: 115070248-115070265	*/- CCTCTTATCC TGGCAGGA	p.402_402del	NM_080687	damaging	P3-low and high
ZMPSTE24	missense	chr1: 40756543-40756543	*/-T	p.C359fs	NM_005857	unknown	P2-high
SF3B1	-	-	-	-	-	-	-
EZH2	-		-	-	-	-	-

Supplementary Table 5 Clinical characteristics of 13 pairs of MDS patients before and after disease progression

No.	Sex	Age	WHO subtype		Blast, %		Karyotype		Progression time
			Before	After	Before	After	Before	After	
P4	F	71	RCMD	RAEB-1	1.0	6.5	complex	complex	21 months
P5	F	34	RCMD	RAEB-1	4.0	7.0	del(5q)	del(5q)	30 months
P6	M	52	RCMD	AML-M2	4.5	48.0	normal	normal	6 months
P7	F	63	RAEB-1	AML-M2	7.0	67.5	del(5q)	del(5q)	4 months
P8	M	77	RARS	RAEB-1	2.0	7.0	del(20q)	del(20q), +8	8 months
P9	M	59	RCMD	AML-M4	4.0	30.5	del(1p)	NA	3 months
P10	F	61	RCMD	RAEB-2	3.0	19.5	complex	complex	28 months
P11	M	59	RCMD	RAEB-1	2.0	7.5	t(1;7)(q11;q11.2),+8	t(1;7)(q11;q11.2),+8	3 months
P12	M	79	RCMD	RAEB-1	4.5	6.8	normal	normal	3 months
P13	M	60	RCMD	AML-M2	1.6	64.0	normal	normal	8 months
P14	M	41	RAEB-1	RAEB-2	5.4	12.6	+8	+8	2 months
P15	M	59	RARS	RAEB-2	1.2	11.6	normal	normal	18 months
P16	M	47	RCMD	RAEB-2	0.4	15.2	normal	normal	5 months

Supplementary Table 6 Mutation analysis in 13 pairs of MDS patients before and after disease progression

Patient No.	Mutated Gene	
	Before	After
**P4**	None	ROBO1
**P5**	DICER1<br>SF3B1<br>ROBO1	DICER1<br>ROBO1
**P6**	DNMT3A	DNMT3A<br>ROBO2
**P7**	ANKRD11<br>DNMT3A	ANKRD11<br>DNMT3A<br>ROBO2
**P8**	TET2<br>SRSF2	TET2<br>SRSF2
**P9**	SRSF2<br>RUNX1<br>WT1<br>DHX9	SRSF2<br>RUNX1<br>WT1
**P10**	SRSF2<br>ANKRD11	SRSF2<br>ANKRD11
**P11**	SRSF2<br>KIF20B	SRSF2<br>KIF20B
**P12**	ASXL1<br>TET2<br>U2AF1<br>UPF3A	ASXL1<br>TET2<br>U2AF1<br>UPF3A<br>STAG2
**P13**	DNMT3A<br>ASXL1	DNMT3A<br>ASXL1
**P14**	ASXL1<br>GATA2<br>U2AF1<br>PTPRD<br>KIF20B<br>DHX9	ASXL1<br>GATA2<br>U2AF1<br>PTPRD
**P15**	None	None
**P16**	None	None

Supplementary Table 7 Clinical characteristics of 193 patients with MDS in targeted sequencing.

Characteristic	Value
**Age, y median (range)**	58 (16-87)
**Male: female, n (%)**	102:91
**Diagnosis, n (%)**	193
RCUD	23 (11.9)
RARS	10 (5.2)
RCMD	65 (33.7)
RAEB1/2	72 (37.3)
RAEB-t	20 (10.4)
CMML	3 (1.6)
**Karyotype**	
normal	108 (56.0)
-5/5q-	9 (4.7)
-7/7q-	10 (5.2)
+8	25 (13.0)
20q-	15 (7.8)
Complex	13 (6.7)
others	13 (6.7)
**Blast percentage in marrow, median (range), %**	6.9±7.8
Neutrophils $<1\times10^9$/l n (%)	1.7±1.9
Median (range)	
Hemoglobin, g/L (mean±SD)	78±29
Platelet,$\times10^9$/L (mean±SD)	89±87
**IPSS-R**	
Lower risk	103 (53.4)
Higher risk	85 (44.0)
Not available	5 (2.6)
**AML transformed, n (%)**	52 (50)
**Median survival**	Not reached

Supplementary Table 8 Characteristics of 26 MDS patients with *ROBO*1/2 mutations

No	Sex	Age	WHO/ FAB subtype	Blast	Karyotype	IPSS-R	ROBO1/2 mutation	Survival (months)	AML transformation
P17	M	83	RARS	1.6	Normal	2.5	ROBO1/p.G145R	22	No
P7	F	63	RAEB-1	7.0	del(5)(q13q33)	5.0	ROBO1/p.R416Q	11+	Yes
P18	M	77	RCMD	3.0	der(7),t(7;17)(q11.1;q12),-13,del(17)inv(17)(p12q11.2)t(7;17)del(7)(q33)	7.5	ROBO1/c.1980+7T>G	12	Yes
P19	M	70	RAEB-1	8.0	del(20q11.2)	5.5	ROBO1/p.R886H	30+	Yes
P4	M	71	RAEB-2	20.0	Normal	5.5	ROBO1/p.S948R	12	Yes
P20	F	58	RCMD	0	Normal	2.5	ROBO1/p.D1135A	44+	No
P21	F	72	RAEB-1	6.1	-2,t(4;11))(p10p10),-10	8.0	ROBO1/p.T1160A	12	Yes
P22	M	79	RAEB-t	25.0	-5,-7,del(12)	9.5	ROBO1/p.T1160A	18	Yes
P23	M	78	RAEB-1	9.4	NA	NA	ROBO1/p.T1160A	9	Yes
P24	F	62	RAEB-1	5.6	Normal	4.5	ROBO1/p.L1171F	17	No
P25	M	45	RAEB-t	10.0	der(3),-5,del(7p15),del(12)(p12),-13,-14,-16,-17,-22	8.5	ROBO1/p.R1144W	41+* (HSCT)	No
P26	M	35	RCMD	2.0	del(17p12)	6.0	ROBO1/p.Q1531P	9	No
P27	M	80	RAEB-1	8.4	+8	6.0	ROBO1/p.S1502N	20+	No
P6	F	52	RAEB-1	7.6	Normal	3.5	ROBO1/p.S1502N	17+	No
P1	F	68	RN	4.5	Normal	2.5	ROBO1/p.R77H	38+	Yes
P5	F	34	RAEB-1	9.0	del(5q13)	4.0	ROBO2/p.G107E	18	Yes
P28	F	65	RCMD	1.5	Normal	4.0	ROBO2/p.N39T	12	No
P29	M	24	RCMD	1.0	Normal	2.0	ROBO2/p.S311G	24+	No
P30	F	39	RCMD	0.6	Normal	4.0	ROBO2/p.S311G	13	No
P31	M	58	RAEB-t	30.0	Normal	5.5	ROBO2/p.R673H	5	Yes
P32	F	55	RARS	3.0	Normal	3.5	ROBO2/p.P1058R	38+	No
P33	M	66	RAEB-1	5.0	Normal	6.0	ROBO2/p.P1058R	10	Yes
P34	M	38	RCMD	4.5	NA	NA	ROBO2/c.3554+6T>-	18	No
P35	M	53	CMML-1	6.4	der(22)[25]	4.5	ROBO2/p.R640H	17	Yes
P3	M	54	RCMD	2.0	Normal	2.5	ROBO2/p.P522Q	24	Yes
P36	M	33	RAEB-1	5.5	Normal	5.0	ROBO2/c.2329-6C>T	65+* (HSCT)	No

Supplementary Table 9 Comparison of clinical characteristics between MDS patients with or without *ROBO* mutations.

Characteristic	*ROBO* mutations (n= 26)	*ROBO* wild type (n= 167)	*P* value
**Age, y median (range)**	58 (24-83)	58 (16-87)	0.293
**Male: female, n (%)**	16:10 (62:38)	86:81 (51:49)	0.340
**Diagnosis, n (%)**			
RCUD	1 (4)	22 (13)	0.213
RARS	3 (12)	7 (4)	0.137
RCMD	6 (23)	59 (35)	0.219
RAEB1/2	11 (42)	61 (37)	0.271
RAEB-t	4 (15)	16 (10)	0.485
CMML	1 (4)	2 (1)	0.354
**Karyotype**			
normal	14 (54)	104 (62)	0.412
-5/5q-	2 (8)	7 (4)	0.604
-7/7q-	3 (12)	7 (4)	0.137
+8	1 (4)	21 (13)	0.320
20q-	1 (4)	14 (8)	0.495
Complex	4 (15)	9 (5)	0.079
**Blast percentage in marrow, mean±SD, %**	6.8±8.8	6.9±7.4	0.940
Neutrophils $<1\times10^9$/l n (%)	1.7±1.9	1.9±2.1	0.740
Median (range)			
Hemoglobin, g/L (mean±SD)	80±22	75±22	0.826
Platelet,$\times10^9$/L (mean±SD)	85±114	90±84	0.826
**IPSS-R**			0.345
Lower risk	11 (42)	92 (55)	0.224
Higher risk	13 (50)	72 (43)	0.511
Not available	2 (8)	3 (2)	-
**AML transformed, n (%)**	13 (50)	39 (23)	0.004
**Median survival**	17.0 months	Not reached	0.017

Supplementary Table 10 Multivariate analysis of survival by Cox Regression

Variable	Uni-variate *P*-Value	Mutivariate *P*-Value	HR	95% CI for HR
Age (<60 versus >60 years)	0.001	0.028	1.664	1.057 – 2.619
Sex (male/female)	0.023	0.272	0.776	0.494 – 1.220
IPSS-R (very low/low/int/high/very high)	0.000	0.000	1.431	1.224 – 1.646
*ROBO*1/2 mutation (WT/mutated)	0.018	0.048	1.801	0.977 – 3.322
DNMT3a mutation (WT/mutated)	0.050	0.605	1.204	0.597 – 2.428
BCOR mutation (WT/mutated)	0.049	0.577	1.329	0.490 – 3.601
TP53 mutation (WT/mutated)	0.019	0.188	0.465	0.148 – 1.455
RUNX1 mutation (WT/mutated)	0.003	0.434	1.427	0.586 – 3.473
SRSF2 mutation (WT/mutated)	0.019	0.061	1.534	0.612 – 3.203
ASXL1 mutation (WT/mutated)	0.762	-	-	
IDH1/2 mutation (WT/mutated)	0.425	-	-	-
U2AF1 mutation (WT/mutated)	0.159	-	-	-
EZH2 mutation (WT/mutated)	0.293	-	-	-
ANRKD11 mutation (WT/mutated)	0.263		-	-
